# Interplay between Cellular Uptake, Intracellular Localization and the Cell Death Mechanism in Triphenylamine-Mediated Photoinduced Cell Death

**DOI:** 10.1038/s41598-020-63991-9

**Published:** 2020-04-23

**Authors:** Rahima Chennoufi, Ngoc-Duong Trinh, Françoise Simon, Guillaume Bordeau, Delphine Naud-Martin, Albert Moussaron, Bertrand Cinquin, Houcine Bougherara, Béatrice Rambaud, Patrick Tauc, Céline Frochot, Marie-Paule Teulade-Fichou, Florence Mahuteau-Betzer, Eric Deprez

**Affiliations:** 1 Laboratory of Biology and Applied Pharmacology (LBPA), CNRS UMR8113, IDA FR3242, ENS Paris-Saclay, Université Paris-Saclay, F-91190 Gif-sur-Yvette, France; 2 UMR9187, CNRS, INSERM, Institut Curie, PSL Research University, Université Paris-Saclay, F-91405 Orsay, France; 3 LRGP, UMR7274 CNRS-Université de Lorraine, F-54000 Nancy, France; 4 Institut Cochin, INSERM U1016-CNRS UMR8104-Université Paris Descartes, Sorbonne Paris Cité, F-75014 Paris, France; 5 Present Address: Institut de Recherches Servier SA, F-78290 Croissy-sur-Seine, France; 6 Present Address: Laboratoire des IMRCP, Université de Toulouse, CNRS UMR5623, Université Toulouse-III - Paul Sabatier, F-31400 Toulouse, France

**Keywords:** Apoptosis, Chemical tools

## Abstract

Triphenylamines (TPAs) were previously shown to trigger cell death under prolonged one- or two-photon illumination. Their initial subcellular localization, before prolonged illumination, is exclusively cytoplasmic and they translocate to the nucleus upon photoactivation. However, depending on their structure, they display significant differences in terms of precise initial localization and subsequent photoinduced cell death mechanism. Here, we investigated the structural features of TPAs that influence cell death by studying a series of molecules differing by the number and chemical nature of vinyl branches. All compounds triggered cell death upon one-photon excitation, however to different extents, the nature of the electron acceptor group being determinant for the overall cell death efficiency. Photobleaching susceptibility was also an important parameter for discriminating efficient/inefficient compounds in two-photon experiments. Furthermore, the number of branches, but not their chemical nature, was crucial for determining the cellular uptake mechanism of TPAs and their intracellular fate. The uptake of all TPAs is an active endocytic process but two- and three-branch compounds are taken up via distinct endocytosis pathways, clathrin-dependent or -independent (predominantly caveolae-dependent), respectively. Two-branch TPAs preferentially target mitochondria and photoinduce both apoptosis and a proper necrotic process, whereas three-branch TPAs preferentially target late endosomes and photoinduce apoptosis only.

## Introduction

Over the past decade, there has been a growing interest in developing and studying two-photon absorption probes for cell imaging and biomedical applications. Indeed, near infrared (NIR) and infrared (IR) wavelengths used for two-photon excitation minimize autofluorescence and maximize tissue penetration due to smaller light scattering/absorption by the tissue and endogenous biomolecules^1^. Such probes were used for several types of applications including fluorescent markers such as nucleus and organelle markers or chemical probes^2–5^. Alternatively, two-photon excitation may allow to trigger photochemical/photobiological processes, for instance in the case of (i) optogenetics for the control of protein activity^6,7^, or (ii) photodynamic therapy (PDT) for light-dependence production of reactive oxygen species (ROS)^8–12^.

**Figure 1:**
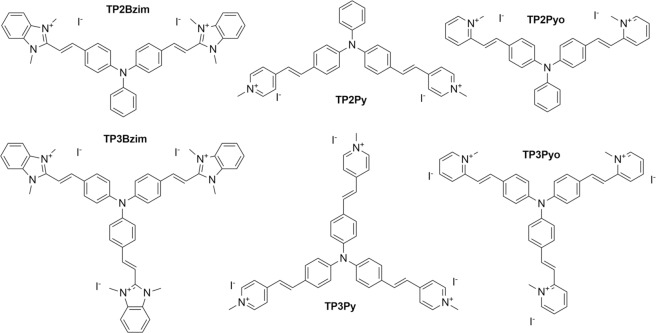
Chemical structures of TPAs.

Vinyl-triphenylamine (TPA) compounds correspond to a family of fluorescent molecules exhibiting high two-photon absorption cross-sections (σ^2^)^13–16^. Their scaffold is comprised of a triphenylamine core bearing two (TP2) or three (TP3) vinyl branches terminated by cationic electron acceptor groups, *e.g*. N-methyl benzimidazolium (Bzim) or pyridinium branched in position para (Py) or ortho (Pyo) with respect to the vinyl bond (Fig. 1). TPAs efficiently bind to the minor grove of AT-rich DNA sequences, leading to a substantial enhancement of their fluorescence quantum yield (Φ_F_). We previously demonstrated that these combined properties of two-photon fluorescence and DNA-binding are particularly suitable for multiphoton imaging of nuclei in fixed cells^13–15,17^.

Recently, novel properties of two compounds, TP2Py and TP3Bzim, were revealed in living cells. In contrast to the observation in fixed cells, the initial localization of TP2Py/TP3Bzim was shown to be exclusively cytoplasmic in living cells^16^. Upon one- (visible light) or two-photon (NIR) excitation, these two compounds triggered cell death, a phenomenon mediated by ROS production and strictly dependent on TPA photoactivation^16,17^. This cell death process is accompanied by a cytoplasm-nucleus relocalization of the TPA fluorescence signal and concomitant morphological hallmarks of apoptosis such as plasma membrane blebbing and cell shrinkage^18^, making them good candidates for application in PDT. These properties were consistently observed in several cell lines, including adherent (HeLa, MCF7, LNCaP, HepG2) and non-adherent (Jurkat) cells^16,17^. Interestingly, a direct nuclear localization of TP2Py/TP3Bzim occurs when apoptosis is caused by another factor than the TPA compound itself, for instance when cells are pre-cultured in the presence of a pro-apoptotic chemical agent (camptothecin)^17^. Thus, TP2Py/TP3Bzim possess the ability to trigger and image apoptosis upon one- or two-photon excitation. However, some differences exist between these two compounds with respect to subcellular localization and the cell death mechanism involved^16^: first, the initial localization (before prolonged light irradiation) of TP2Py was mainly mitochondrial, whereas TP3Bzim was found mainly in late endosomes and also in mitochondria, but to a lesser extent than TP2Py. For both compounds, their final nuclear localization accounts for subcellular (organelle) damages related to photoinduced apoptosis. Second, if TP2Py/TP3Bzim primarily induce apoptosis at short time scales, TP2Py, but not TP3Bzim, also induces a proper necrotic effect.

Taking into account that the structures of TP2Py/TP3Bzim differ by both the number of branches and the chemical nature of their electron acceptor group, we extended in the present study the characterization of TPAs to a set of analogues (TP3Py, TP2Bzim, TP2Pyo and TP3Pyo; Fig. 1) that will allow discerning the influence of the number of branches and the terminal cationic moiety on the compound subcellular fate. We assessed first the ability of these analogues to trigger cell death upon one- or two-photon excitation and further studied the cellular uptake mechanism of the three pairs of TPAs to explore the interplay between cell entry, intracellular localization and photoinduced cell death mechanism. We found that the chemical nature of the electron acceptor group is critical for the overall cell death efficiency, with TP-Pyo derivatives being much less efficient than TP-Bzim or TP-Py derivatives, regardless of the excitation mode (one- or two-photon), whereas the number of branches primarily determines the cell uptake pathway and subsequent intracellular properties, *i.e*. subcellular localization, ROS generation efficiency and cell death mechanism.

## Results and Discussion

### *In vitro* absorption and fluorescence properties of TPAs

The spectroscopic characteristic values of TP3Py, TP2Bzim, TP2Pyo and TP3Pyo (λ_abs/exc,1-hν_, λ_exc,2-hν_, λ_em_, Φ_F_, σ^2^) are reported in Supplementary Table S1 and compared to the previously characterized TP2Py and TP3Bzim compounds. All TPAs display maximum visible absorption/excitation wavelengths (λ_abs/exc,1-hν_) in the 433–477 nm range with very poor fluorescence emission when free in aqueous buffer. DNA-binding leads to a red-shifted λ_abs/exc,1-hν_ (457–506 nm) and a substantial increase of fluorescence emission. However, the Φ_F_ values significantly differ from one compound to another, with the chemical nature of the electron acceptor group being determinant (Bzim >> Pyo ≥ Py). For each pair, the 2-branch compound has consistently a higher Φ_F_ value than the 3-branch compound (0.54 and 0.34 for TP2Bzim and TP3Bzim, respectively; 0.17 and 0.08 for TP2Pyo and TP3Pyo, respectively; 0.08 and 0.01 for TP2Py and TP3Py, respectively) that is explained by the extrahelical position of the third branch when the TPA is bound to the DNA minor groove^15^. For all compounds, except TP2Py, the DNA-dependent increase in the Φ_F_ value is accompanied by a blue-shift of the maximum emission wavelength (λ_em_) with the same hierarchy: Bzim (Δλ_em,TP3Bzim_ = 48 nm; Δλ_em,TP2Bzim_ = 31 nm)> Pyo (Δλ_em,TP3Pyo_ = Δλ_em,TP2Pyo_ = 27 nm)> Py (Δλ_em,TP3Py_ = 15 nm; Δλ_em,TP2Py_ = −4 nm). Again, the nature of the electron acceptor group determines the emission wavelength: Bzim (λ_em,TP3Bzim_ = 572 nm; λ_em,TP2Bzim_ = 576 nm) <Pyo (λ_em,TP3Pyo_ = 611 nm; λ_em,TP2Pyo_ = 610 nm) <Py (λ_em,TP3Py_ = 641 nm; λ_em,TP2Py_ = 637 nm).

Regarding the two-photon absorption/fluorescence properties, all TPAs display high σ^2^ values comprised between 325 and 764 GM and maximum NIR excitation wavelengths (λ_exc,2-hν_) in the 750–880 nm range, depending on the compound. By contrast to what observed for the Φ_F_ value, the σ^2^ value in each pair is higher for the 3-branch (octupolar arrangement) than for the 2-branch (quadrupolar arrangement) compound: 764 and 495 GM for TP3Bzim and TP2Bzim, respectively; 388 and 365 GM for TP3Pyo and TP2Pyo, respectively; 500 and 325 GM for TP3Py and TP2Py, respectively. However, taking into account that the σ^2^ values are less influenced by the nature of electron acceptor group, the hierarchy of *in vitro* two-photon brightness (σ^2^xΦ_F_) principally follows the hierarchy based on the Φ_F_ value: 267 and 258 GM for TP2Bzim and TP3Bzim, respectively; 62 and 31 GM for TP2Pyo and TP3Pyo, respectively; 26 and 5 GM for TP2Py and TP3Py, respectively.

### Photoinduced effects of TPAs in living cells

#### One-photon photoactivation

**Figure 2:**
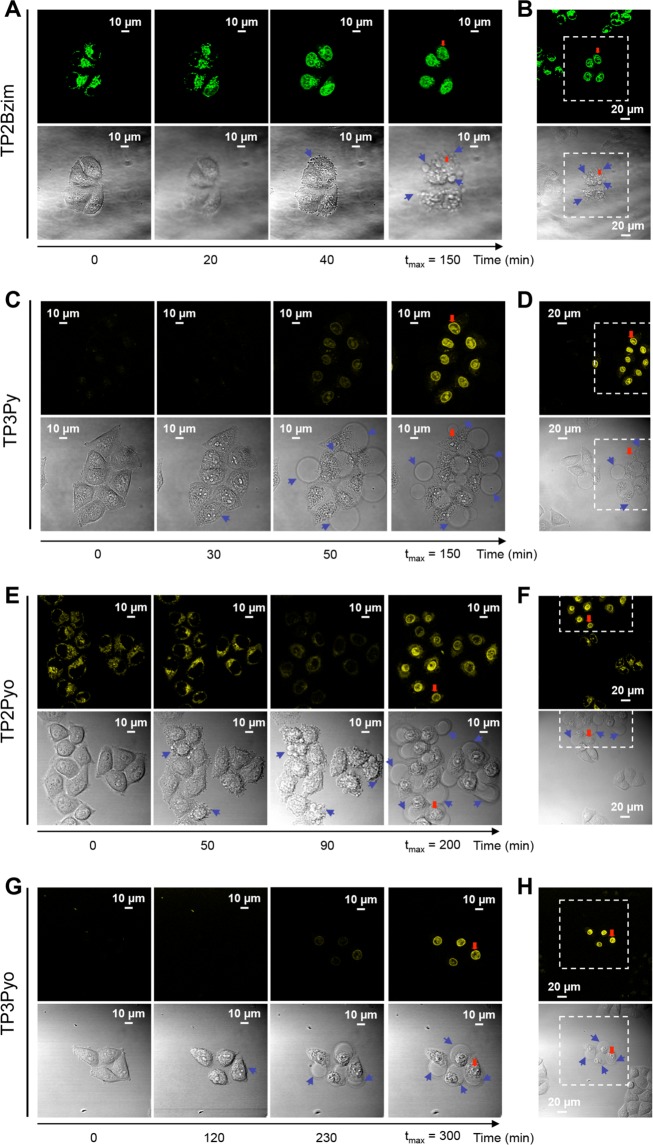
Confocal fluorescence imaging of TPA-treated living cells upon prolonged visible light irradiation. HeLa cells were pre-incubated with 2-µM TPA (**A,B**: TP2Bzim; **C,D**: TP3Py; **E,F**: TP2Pyo; **G,H**: TP3Pyo) for 2 h at 37 °C before continuous irradiation (458 nm; irradiance, 30 mW/cm^2^) (see Methods for emission slit settings). For each compound, the image series show observations at four relevant times (a more complete view is shown in Supplementary Fig. S1): the initial observation (time 0), the beginning of the nuclear translocation of TPA fluorescence, the appearance of plasma membrane blebs (indicated by blue arrows in DIC – differential interference contrast – transmission images) and the time of maximum fluorescence intensity in nuclei (t_max_). Enlarged field of observation is shown at t_max_ for each TPA (panels B, D, F, H), showing that only cells illuminated at t = 0 are characterized by both TPA nuclear translocation and membrane blebbing. Cells outside the irradiated area (delineated by dashed lines) display normal morphologies and cytoplasm-located fluorescence. Cells indicated by a red arrow serve as a landmark.

Living HeLa cells, treated with TP3Py, TP2Bzim, TP2Pyo or TP3Pyo and irradiated by visible light (458 nm), displayed similar features than those previously reported for TP3Bzim and TP2Py^16,17^. All cells displayed an initial localization of the TPA fluorescence signal which is cytoplasmic (Fig. 2). Under prolonged illumination, we consistently observed a nuclear translocation of the fluorescence signal with the beginning of this translocation occurring on a time scale of 20–230 min (after the start of illumination), depending on the compound. These values for TP2Bzim (20 min) and TP3Py (50 min) remain comparable with those characterizing TP3Bzim and TP2Py (20 and 40 min, respectively)^17^, although TP2(3)Pyo led to slower kinetics (>90 min). The stronger fluorescence emission in the nucleus compared to the initial cytoplasmic emission is in agreement with *in vitro* studies showing the large fluorescence enhancement of TPAs upon DNA-binding (Supplementary Table S1). As previously found with TP3Bzim/TP2Py, the nuclear translocation of the four new analogues coincides with both cell shrinkage and the appearance of plasma membrane blebs (see images in the transmission mode on Fig. 2), two morphological hallmarks of cell apoptosis^18^. Of note, both phenomena, *i.e*. the TPA relocalization to the nucleus and membrane blebbing, were strictly dependent on the photoactivation process since only cells in the illuminated area displayed (i) a strong nuclear fluorescence signal and (ii) a dramatic morphological change (Fig. 2, panels B, D, F, H). The cells outside this area displayed a constant cytoplasmic location of fluorescence and a normal morphology. These results obtained with the new analogues indicate that all TPAs share – at least qualitatively – similar pro-apoptotic properties upon prolonged visible light irradiation.

#### Two-photon photoactivation

**Figure 3:**
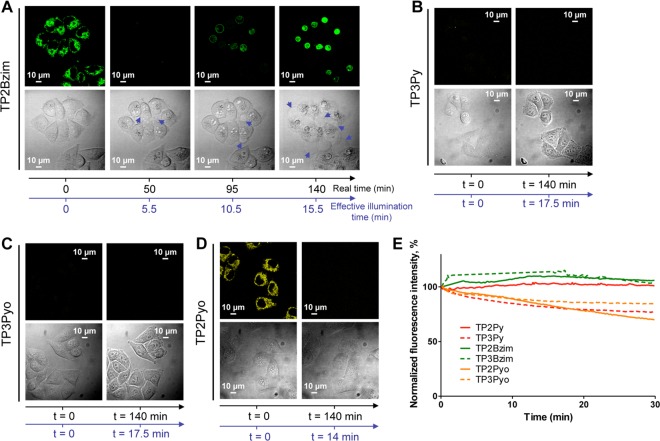
Two-photon TPA excitation in living cells. HeLa cells were pre-incubated with 2-µM TPA for 2 h at 37 °C before two-photon excitation at 830 nm for TP2Bzim (**A**), TP3Py (**B**), TP3Pyo (**C**) or 850 nm for TP2Pyo (**D**) (irradiance, 1.25 W/cm^2^) (see Methods for emission slit settings). Plasma membrane blebs are indicated in DIC transmission images by blue arrows. The time scale corresponds to the real time (black axis) or the effective illumination time accounting for the z-stack as explained in Methods (blue axis) (number of slices: 8 for TP3Py and TP3Pyo; 9 for TP2Bzim; 10 for TP2Pyo). (**E**) Photobleaching properties of TPAs under continuous two-photon irradiation. TPAs were subjected to prolonged NIR irradiation and the normalized fluorescence intensity was plotted as a function of irradiation time. The two-photon fluorescence was measured *in vitro* as described in Methods. λ_exc,2-hν_: 760 nm (TP3Bzim), 830 nm (TP2Bzim, TP3Py and TP3Pyo), 850 nm (TP2Pyo), 860 nm (TP2Py).

Two-photon experiments in living HeLa were performed in a similar manner than one-photon experiments except that each TPA was excited by its optimal two-photon excitation wavelength (Fig. 3A–D). Conversely to one-photon experiments, the new analogues clearly display differential responses regarding their ability to induce cell death upon two-photon excitation. Only cells treated with TP2Bzim underwent significant apoptosis with a concomitant nuclear translocation of fluorescence (Fig. 3A), in a similar manner to that previously observed with TP3Bzim/TP2Py^16^. By contrast, no nuclear translocation or morphological change of treated cells were observed with TP3Py, TP3Pyo or TP2Pyo (Fig. 3B–D). Their fluorescence signals were low or continuously decreased without nuclear relocalization, suggesting that photobleaching preferentially occurs for these compounds under continuous two-photon illumination. The susceptibility of TPAs to photobleaching under two-photon illumination was then measured *in vitro* using a FCS (fluorescence correlation spectroscopy) set-up as described in Methods. *In vitro* results show a continuous decrease of fluorescence intensities for TP3Py, TP2Pyo and TP3Pyo, while fluorescence intensities remained stable for TP3Bzim, TP2Bzim and TP2Py (Fig. 3E), in agreement with observations in the cell context. Altogether, these results indicate that, in contrast to TP3Bzim, TP2Bzim and TP2Py, the three other compounds are unable to induce a two-photon-dependent cell death process, most likely for reasons related to faster photodamage.

### Structural determinant of the initial subcellular TPA localization

**Figure 4:**
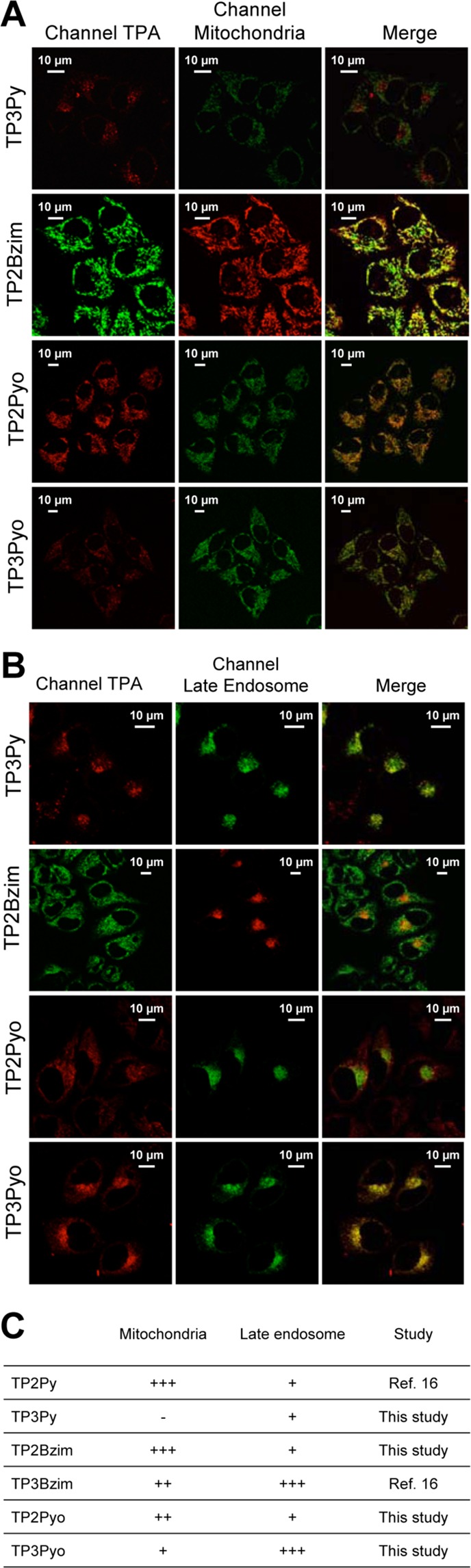
Colocalization of TPAs with mitochondria (**A**) or late endosomes (**B**) in HeLa cells before light-induced nuclear translocation. Left, imaging channels of TPAs. Middle, imaging channels of organelle trackers. Right, corresponding merged images. Yellow-to-orange areas indicate TPA/organelle colocalization. Details about TPA concentrations, organelle trackers, excitation/emission settings used in confocal microscopy are provided in Supplementary Methods. Colocalization results for all TPAs are summarized in panel (**C**).

All cationic TPAs presented in Fig. 1 show an exclusive cytoplasmic localization before light irradiation and subsequent nuclear translocation. The global positive charge (2^+^ to 3^+^ for 2- and 3-branch compounds, respectively) is important for the cytoplasmic retention. Accordingly, we have previously shown that living cells treated with a neutral TPA displayed a non-specific and homogeneous staining pattern, with both cytoplasmic and nuclear staining, even before prolonged illumination^17^. Nevertheless, the fluorescence signal pattern of cationic TPAs in the cytoplasm differs between compounds. Indeed, we have previously shown that TP2Py mainly colocalized with mitochondria (together with a slight localization in late endosomes that was consistently observed) while TP3Bzim only partially colocalized with mitochondria^16^. For TP3Bzim, most of the colocalization signal originated from late endosomes. Thus, we extended here the colocalization study to the other compounds to identify the structural features of TPAs that govern their intracellular fate. The colocalization of TP2Bzim, TP3Py, TP2Pyo and TP3Pyo with either mitochondria or late endosomes was then investigated in HeLa cells. Although all TPAs (except TP3Py) displayed a dual location, the strongest colocalization signals were obtained with mitochondria for 2-branch derivatives (TP2Bzim and TP2Pyo) and with late endosomes for 3-branch derivatives (TP3Py and TP3Pyo) (Fig. 4). Altogether, our results show that the number of branches, rather than the chemical nature of the electron acceptor group, is critical to determine the subcellular TPA localization with 2- and 3-branch derivatives preferentially targeting mitochondria and late endosomes, respectively.

### Relationship between the subcellular TPA localization and the subsequent photoinduced cell death mechanism

**Figure 5:**
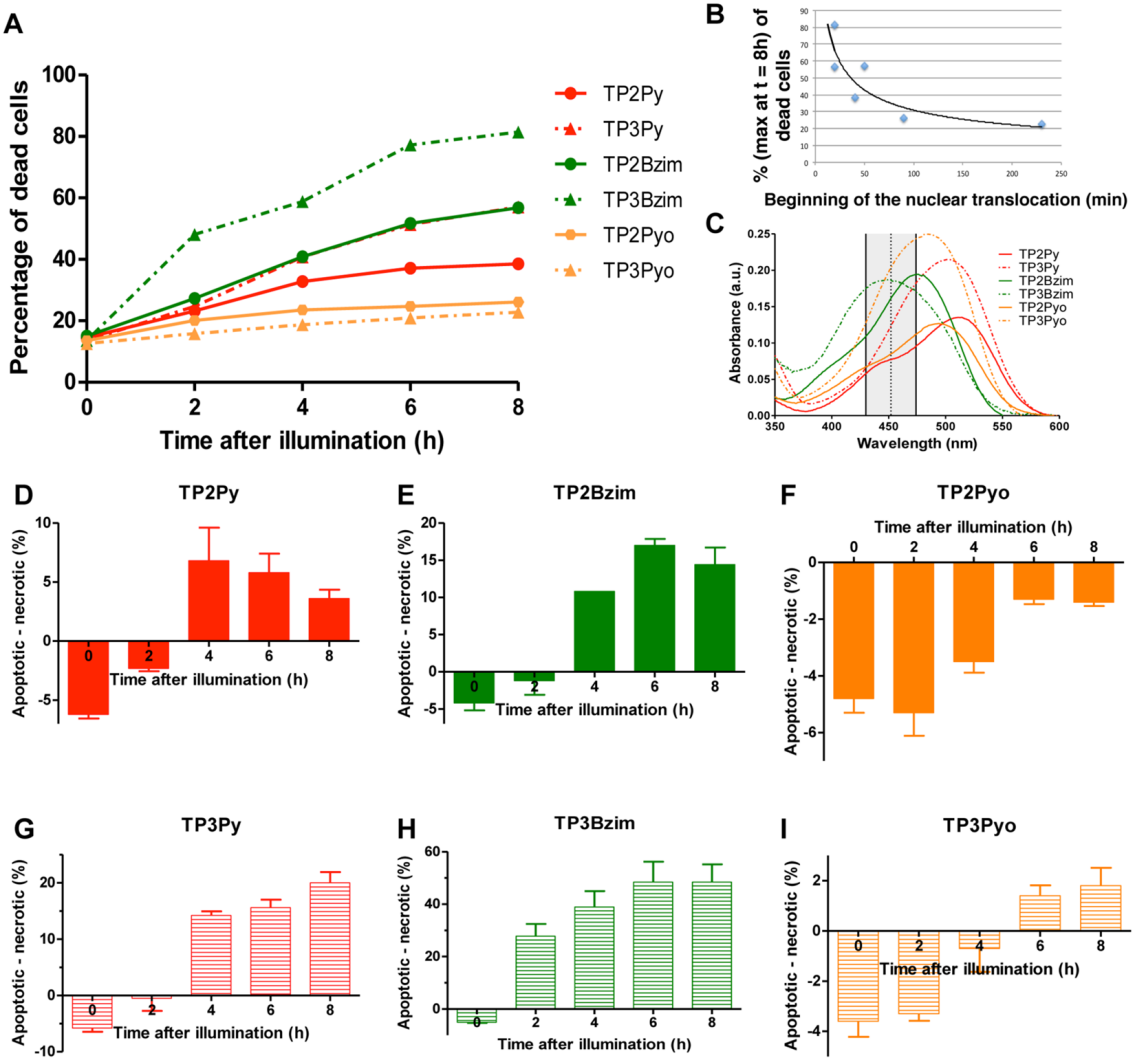
Flow cytometric analysis of apoptotic/necrotic subpopulations of cells upon TPA treatment and light illumination. Living Jurkat cells were pre-treated with 2-µM TPA for 2 h and subjected to light illumination (5 mW.cm^−2^) for 30 min (Mercury lamp: 130 W; 380–600 nm + filter: 452 ± 22.5 nm). Treated cells were then incubated for various times in the dark at 37 °C before Annexin V/DAPI treatment and analysis. (**A**) Total percentages of dead cells (Annexin V^+^/DAPI^+/−^) as a function of time after illumination. The plot shows representative data of 3 independent experiments for each TPA. (**B**) Relationship between the maximum percentage of dead cells (at t = 8 h) and the beginning of TPA nuclear translocation (see Fig. 2). (**C**) Corresponding absorption spectra of TPAs (+DNA). Also shown is the transmission profile of the excitation filter (in grey) used in flow cytometry experiments. (**D–I**) Apoptotic cell subpopulation (Annexin V^+^/DAPI^−^) minus necrotic subpopulation (Annexin V^+^/DAPI^+^) for each TPA as a function of time after illumination (corrected by apoptotic/necrotic subpopulations measured in the absence of illumination). The bar graphs show mean ± SD values from 3 independent experiments. Related two-dimensional flow cytometry dot plots, control experiments (+/− TPA, + /− illumination) and kinetics of apoptosis/necrosis are shown in Supplementary Figs. S2, S3.

Phosphatidylserine (PS) recognition by Annexin V is commonly used to probe dead cells: (i) apoptotic cells which are characterized by PS externalization occurring at the plasma membrane but also (ii) necrotic cells with damaged plasma membranes allowing Annexin V staining even if PS remains on the inner face of the membrane^16,19^. The use of 4’,6’-diamino-2-phenylindole (DAPI) in combination with Annexin V allows to differentiate between the two subpopulations of dead cells since DAPI does not permeate cells with intact plasma membrane^16^. Thus, the double Annexin V/DAPI treatment followed by flow cytometer analysis allows discrimination of living (Annexin V^−^/DAPI^−^), apoptotic (Annexin V^+^/DAPI^−^) and necrotic cells (Annexin V^+^/DAPI^+^). To address the precise TPA-induced cell death mechanism, non-adherent Jurkat cells were pre-treated with TPAs, subjected to light illumination and analysed by flow cytometry just after 30 min-illumination (= time 0) and at various times after illumination, up to 8 h (Fig. 5 and Supplementary Fig. S2).

First, the overall kinetics of cell death (apoptotic + necrotic cells) show that TP2(3)Pyo are much less efficient than other compounds for triggering cell death upon photoactivation (Fig. 5A). For other compounds, TPAs are efficient in the following order: TP3Bzim > TP2Bzim = TP3Py > TP2Py. Their ability to induce cell death is related to the kinetics of TPA nuclear translocation after photoactivation (Fig. 5B), confirming that the nuclear relocalization process, as observed in fluorescence images of irradiated TPA-treated cells, accounts for the beginning of the cell death process. Note that no simple relationship exists between absorption properties of TPAs (relative to the band-pass filter used in flow cytometry experiments: TP3Pyo > TP3Bzim > TP2Bzim > TP3Py > TP2Pyo > TP2Py) (Fig. 5C) and their ability to trigger cell death, although the nature of the electron acceptor group appears to be critical for the overall cell death efficiency with the following hierarchy: Bzim> Py >> Pyo, involving other properties (not mutually exclusive) such as ROS generation or TPA cellular uptake.

We next quantified at various times after illumination the two subpopulations of dead cells. For each TPA, the difference between apoptotic and necrotic cells is reported as a function of time in Fig. 5D–I. At time 0, the necrotic cell subpopulation was consistently more represented than the apoptotic cell subpopulation, accounting for a small population of apoptotic cells already present before illumination (as observed in TPA-treated cells without illumination; Supplementary Fig. S3) for which the apoptosis->late apoptosis/necrosis transition is accelerated upon illumination. All kinetic profiles show first a significant increase in the apoptotic subpopulation, regardless of the TPA, which is also strictly dependent on illumination (compare with control experiments: without illumination +/−TPA; Supplementary Fig. S3). However, if TP3Py, TP3Bzim and TP3Pyo displayed a continuous increase in the apoptotic population, 2-branch TPAs displayed a distinct profile with the presence of a second phase where the necrotic subpopulation becomes significantly more represented than the apoptotic subpopulation. Importantly, such a differential qualitative behaviour in terms of cell death mechanism is independent of the overall cell death kinetic, suggesting that the Annexin V^+^/DAPI^+^ subpopulation of TP2-treated cells is more related to a necrotic process than to a progression of apoptotic cells toward late apoptosis. Such a differential behaviour was already suggested for the TP3Bzim/TP2Py pair^16^. Beyond the confirmation that light irradiation may trigger different cell death mechanisms among the TPA compound family, our results show that, as observed for the subcellular TPA localization, the number of branches is the main structural feature accounting for the apoptosis-necrosis balance upon light irradiation. All TPAs primarily induce apoptosis but 2-branch compounds also induce a proper necrotic process. Altogether, our results suggest that the subcellular localization of TPAs influences the precise mechanism of cell death involved (apoptosis *vs* necrosis) upon photoactivation.

### Intracellular ROS generation upon TPA photoactivation

**Figure 6:**
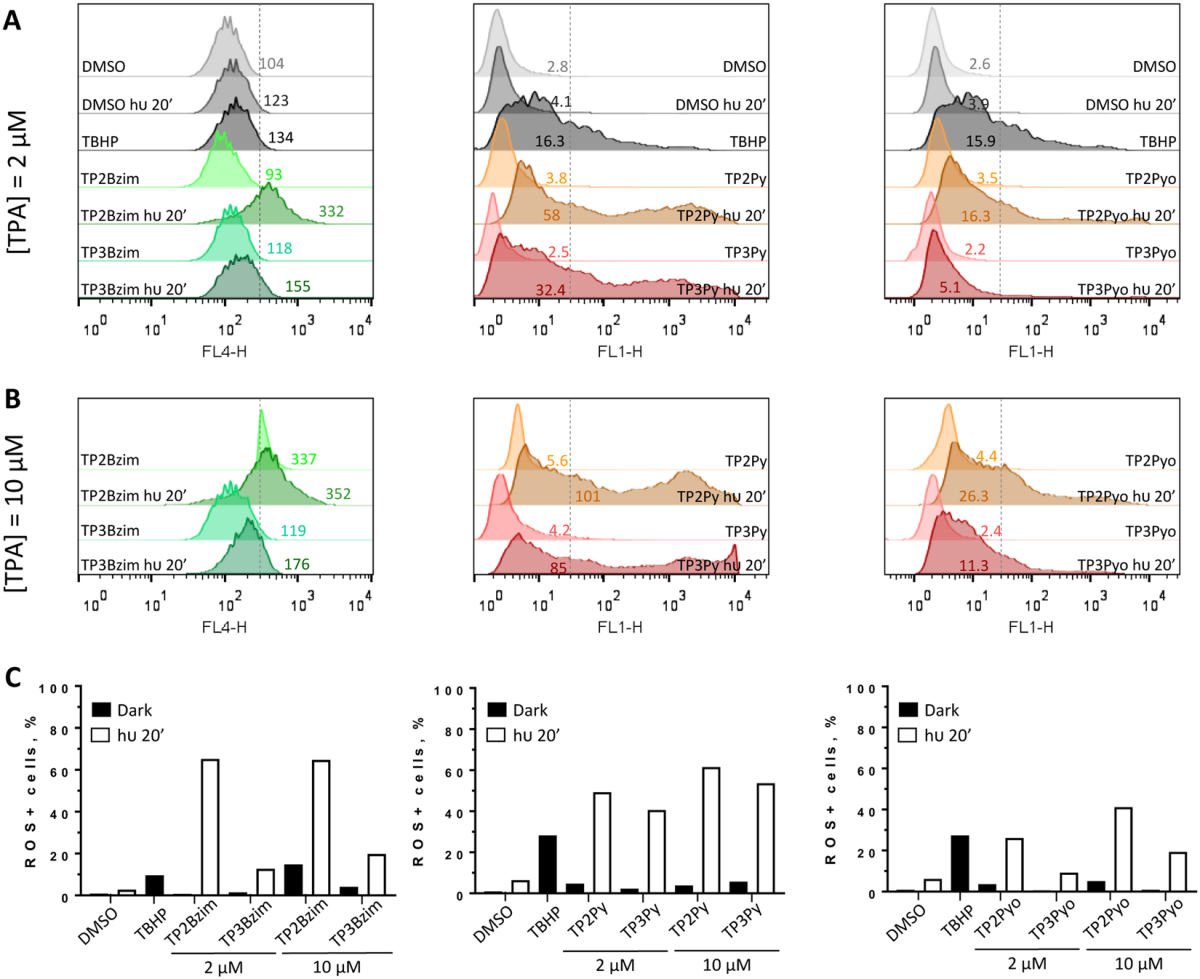
Flow cytometric analysis of intracellular ROS detection upon TPA photoactivation. (**A-B**) modal representations of the number of cells as a function of the fluorescence emission intensity of ROS detectors. HeLa cells were treated with 2 µM (**A**) or 10 µM (**B**) of TPA compounds and then subjected or not to light irradiation for 20 min before treatment with either the ROS Deep Red dye (FL4 channel; left panel) or H_2_DCF-DA (FL1 channel; middle and right panels) (depending on the emission wavelength of TPAs) and flow cytometric analysis (see Methods). Cells treated with DMSO alone (1‰) +/− light or TBHP (100 µM) for 1 h were used as negative and positive controls, respectively. Light-treated samples are explicitly mentioned in figures (= hν 20’). The indicated numbers correspond to geometric mean values of fluorescence intensities. The vertical dotted lines represent the limits between negative and positive cells in terms of fluorescence (named thereafter L) by taking the “DMSO/dark” condition as a reference. The percentages of positive cells were estimated based on the L value (= [number of cells _(fluorescence intensity> L)_] × 100/[total number of cells]) and are shown in histograms (**C**).

The photoinduced TPA-mediated production of ROS was measured first in the cell context by flow cytometric analysis, using two fluorescent ROS sensors that exhibit emission enhancement in the presence of ROS. For spectral reasons, we used the ROS detector 2’,7’-dichlorodihydrofluorescein diacetate (H_2_DCF-DA)^20,21^ for TP2(3)Py and TP2(3)Pyo compounds and the ROS Deep Red dye^22^ for TP2(3)Bzim compounds (Fig. 6). H_2_DCF-DA is a non-specific probe for reactive oxygen/nitrogen species, mainly hydroxyl radical (^•^OH) and peroxynitrite (ONOO^−^) (also indirectly other species in the cell context such as hydrogen peroxide (H_2_O_2_) and superoxide ion (O_2_^•−^)), whereas the Deep Red dye preferentially detects ^•^OH and O_2_^•−^. All TPAs (used at 2 or 10 µM) efficiently induced intracellular ROS production in a strict light-dependent manner (except TP2Bzim when used at 10 µM that also induced significant ROS production in non-irradiated cells). Except TP2(3)Pyo which are less efficient than other compounds, 2 µM of TP2(3)Py or TP2(3)Bzim were more efficient for inducing ROS than 100 µM of the commonly used oxidative stress inducer Tert-butyl-hydroperoxide (TBHP)^23–25^. TP2(3)Pyo compounds which are less efficient to photoinduce cell death compared to TP2(3)Py and TP2(3)Bzim (Figs. 2 and 5), are also less efficient for ROS generation.

To note, the ability of TPAs to efficiently photoinduce intracellular ROS production is not predictable by their photophysical properties as measured *in vitro*. Indeed, all TPAs have singlet oxygen (^1^O_2_) quantum yield close to 0 (Φ_Δ_ ≤ 0.01; Supplementary Table S1 and Fig. S4A). However, they can photoinduce weak production of O_2_^•−^ and ^•^OH, as measured with the Diphenylisobenzofuran (DPBF)^26–28^ and Aminophenyl fluorescein (APF)^29,30^ probes which are selective for O_2_^•−^/^1^O_2_ and ^•^OH/^1^O_2_, respectively (Supplementary Fig. S4B,C). Such a weak production via photochemical reaction cannot explain by itself the large amount of ROS detected in the cell context, but could be sufficient to initiate mitochondrial permeability transition that, in turn, amplifies ROS production, in accordance with the observed protection effect of cyclosporine A, an inhibitor of the mitochondrial permeability transition, on the TPA-mediated ROS production^17^. Furthermore, from the present flow cytometry data as shown in Fig. 6, for each TPA pair (TP-Bzim, TP-Py and TP-Pyo), the 2-branch compound was systematically more efficient for ROS production than its 3-branch counterpart, which is consistent with the preferential mitochondrial localization of 2-branch TPAs and probably also explains their specific effect on necrosis. Nevertheless, the number of branches is not determinant for the overall cell death efficiency which primarily relies on the chemical nature of their electron acceptor group as mentioned above (Bzim > Py >> Pyo), the exact reason is not clear but may be due to more subtle considerations about the spatio-temporal intracellular distribution of TPAs that influences both the timing and subcellular localization of ROS generation.

### Relationship between the cellular uptake mechanisms and the intracellular fates of TPAs

**Figure 7:**
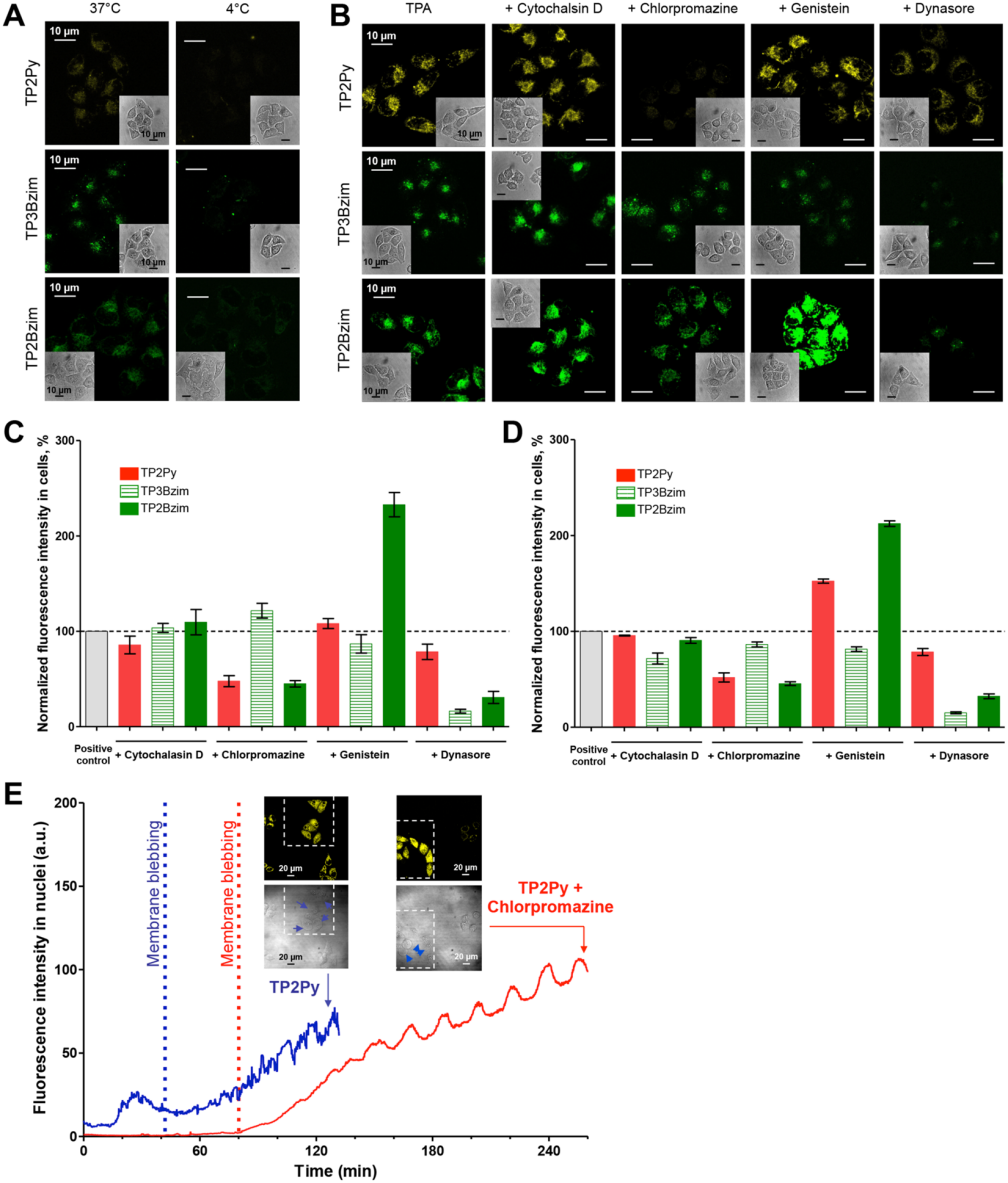
Determination of TPA cellular uptake mechanism. (**A**) Energy dependence of TPA uptake: HeLa cells were incubated with TPAs at 37 °C (left) or 4 °C (right) and analysed by fluorescence imaging. (**B–D**) Mechanisms of endocytosis: HeLa cells were incubated with TPAs at 37 °C in the presence of various endocytosis inhibitors (left: positive control without inhibitor) and analysed by fluorescence imaging (**B,C**) or flow cytometry (D) (see Methods for experimental details). In (**A,B**), fluorescence images are shown for each TPA (top: TP2Py; middle: TP3Bzim; bottom: TP2Bzim) with corresponding DIC images (insets). In (**C,D**), quantitative analysis of TPA fluorescence intensity is shown for inhibitor-treated cells (normalized by the intensity level obtained without inhibitor). Results shown in panels B–D correspond to a TPA-incubation time of 90 min. Except the “TP3Bzim + genistein” condition, similar results were obtained with TPA-incubation times of 30 or 60 min (not shown). Results corresponding to the “TP3Bzim + genistein” condition with incubation times of 30 or 60 min are shown in Supplementary Fig. S5. The bar graphs in panels C,D show mean ± SD values from 3 independent experiments. (**E**) Kinetics of nuclear accumulation of TP2Py in HeLa cells treated (red) or not (blue) with chlorpromazine and subjected to continuous light illumination (light illumination conditions are similar to those described in the Fig. 2 legend). Results show that chlorpromazine delays both TPA nuclear translocation (∆_translocation_ ≈ +45 min) and membrane blebbing (∆_blebbing_ ≈ +40 min; vertical dashed line indicates the appearance of first membrane blebs). Fluorescence/DIC images are also shown at the end of the process and confirm that only cells in the irradiated area (delineated by dashed lines) display both a nuclear translocation of the TPA emission signal (fluorescence image) and membrane blebbing (blue arrows in DIC image).

In order to determine first whether TPA uptake is an active (energy-dependent endocytosis) or passive (energy-independent) process^31,32^, HeLa cells were incubated with TPAs at 37 °C (normal conditions) or 4 °C (active processes are blocked). For quantification reasons, the most fluorescent TPAs (TP2Py, TP3Bzim and TP2Bzim) were compared (Fig. 7A). Lowering the temperature led to a near complete inhibition of TPA uptake, regardless of the compound, demonstrating that TPA uptake occurs exclusively via endocytosis.

In order to determine the endocytic pathway^33^ involved in TPA uptake, cells were pre-treated (30 min-incubation) with various endocytosis inhibitors before addition of TPA (additional 90 min-incubation) and analysed by fluorescence microscopy (Fig. 7B,C) or flow cytometry (Fig. 7D). Chlorpromazine that specifically inhibits clathrin-dependent endocytosis^31,34^ had slight or no effect on TP3Bzim uptake whereas TP2Py/2Bzim uptakes were significantly affected. Consistent with the inhibition of TP2Py/2Bzim uptake by chlorpromazine, we also observed a significant inhibition of the uptake of TP2 compounds by dynasore, an inhibitor of dynamin which is involved in clathrin-dependent endocytosis^33,35^. TP3Bzim uptake which primarily corresponds to a clathrin-independent mechanism was even more sensitive to dynasore, suggesting the involvement of caveola-dependent endocytosis which is a clathrin-independent and dynamin-dependent process^33,35–39^. Accordingly, a slight but significant inhibition effect of genistein, a tyrosine kinase inhibitor that disrupts caveola-related endocytosis^40,41^, was observed on TP3Bzim uptake (after 90 min-incubation of TPAs) but not on TP2 compounds. This inhibition was even more pronounced after shorter times of incubation, 30 or 60 min (Supplementary Fig. S5). This suggests that TP3Bzim uptake occurs predominantly via a caveola-dependent endocytosis mechanism. However, the decrease in the genistein-mediated inhibition of TP3Bzim uptake as a function of time indicates a compensatory effect through the upregulation of a clathrin- and caveola-independent mechanism. It is worth noting that the mechanism by which genistein enhances cell uptake of TP2Py/2Bzim could correspond to a related phenomenon although the precise mechanism is still unclear and requires further investigations. Treatment with cytochalasin-D, an inhibitor of F-actin elongation involved in macropinocytosis^31^, had no effect on TP2 compounds uptake. Although cell-imaging data did not show significant effect of cytochalasin-D on TP3Bzim uptake, a moderate but reproducible inhibition effect was observed in flow cytometry experiments, characterized by a better statistical significance (30,000 and 50 cells analyzed in flow cytometry and microscopy experiments per condition, respectively). Alternatively, and not mutually exclusive with the caveola-dependent endocytosis, the clathrin- and dynamin-independent macropinocytosis process^33,35,38,42,43^ could be also involved in TP3Bzim uptake. In this context, the dynasore-mediated inhibition of TP3Bzim uptake could be also related to a previously described dynamin-independent inhibition mechanism of dynasore on macropinocytosis^35^. As expected, all efficient inhibitors (chlorpromazine for TP2Py (Fig. 7E) and TP2Bzim (Supplementary Fig. S6A); genistein for TP3Bzim (Supplementary Fig. S6B)) delayed the appearance of membrane blebs and the concomitant TPA nuclear translocation, corresponding in each case to a direct effect on the intracellular TPA concentration since no qualitative influence of endocytosis inhibitors was observed on the subcellular localization of TPAs (Supplementary Fig. S7).

## Conclusion

All TPA compounds tested in living cells, TP2(3)Py, TP2(3)Bzim and TP2(3)Pyo, led to cell death upon one-photon excitation, albeit much less efficiently for the Pyo subfamily. Despite poor ROS quantum yields (^1^O_2_, O_2_^•-^, ^•^OH) as measured *in vitro*, the apparent PDT effect of photoactivated TPAs in the cell context mainly relies on high amplification of endogenous ROS production, due at least in part to their mitochondrial targeting properties. Regarding two-photon photoactivation, only TP2Py and TP2(3)Bzim efficiently triggered cell death, making them good candidates for two-photon PDT. Other compounds, TP3Py and TP2(3)Pyo, were found to be inefficient to trigger cell death upon two-photon excitation. Besides the nature of the electron acceptor group which already quantitatively influences the overall photoinduced cell death efficiency in one-photon experiments (TP-Bzim > TP-Py >> TP-Pyo), such inefficiency seems to be also related to susceptibility to photobleaching in two-photon experiments, more than to proper two-photon absorption properties (there is no correlation between the ability of TPAs to induce cell death upon two-photon excitation and their σ^2^ values). Furthermore, we found that some differences exist between TPAs with respect to the precise cell death mechanism involved upon photoactivation. Clearly, the number of vinyl branches, but not the chemical nature of the acceptor group, plays a key role in determining the cell death mechanism with 2-branch TPAs inducing a proper necrotic process whereas all TPAs induce apoptosis. Such a qualitative and mechanistic difference is related to the initial subcellular TPA localization (before photoactivation) which itself is related to the cellular uptake mechanism. 2-branch compounds are taken up via clathrin-dependent endocytosis and preferentially target mitochondria whereas 3-branch compounds are taken up via clathrin-independent endocytosis (predominantly caveolae-dependent, alternatively macropinocytosis) and preferentially target late endosomes. Although this structure-activity relationship of TPAs is clear, further studies are required to precisely explain the interconnection between the endocytosis pathway, the subcellular localization and the cell death mechanism involved.

## Methods

### Synthesis of TPAs

Synthesis of TP2(3)Py and TP2(3)Bzim was performed as previously described^13,15^. Synthesis of TP2(3)Pyo is described in Supplementary Methods. ^1^H and ^13^C NMR spectra are shown in Supplementary Fig. S8.

### Spectroscopic characterization

Absorption and emission spectra of TPAs (+/−DNA) were measured on a Uvikon XL spectrophotometer (Secomam) and a Cary-Eclipse spectrofluorimeter (Varian), respectively. Two-photon excitation spectra were measured using a home-built set-up equipped with a 80-MHz mode-locked Mai-Tai^®^ Ti-Sapphire tunable laser (690–1040 nm, 100-fs laser pulse; Spectra Physics) as previously described^16,44^. More details about experimental conditions for the determination of spectroscopic/fluorescence parameters are reported in the legend of the Supplementary Table S1.

### Photophysical properties: *In vitro* measurements of ^1^O_2_, O_2_^·-^ and ^•^OH generation upon light excitation of TPAs

The absorption spectra were recorded on a UV-3600 UV–Vis double beam spectrophotometer (Shimadzu). The fluorescence spectra were recorded on a Fluorolog FL3–222 spectrofluorimeter (HORIBA Jobin Yvon) equipped with a 450 W Xenon lamp, a thermo-stated cell compartment (25 °C), a UV–Vis photomultiplier R928 (Hamamatsu), and an InGaAs infrared detector (DSS-16A020L Electro-Optical System Inc). The excitation beam was diffracted by a double ruled grating SPEX monochromator (1200 grooves/mm blazed at 330 nm). ^1^O_2_ emission was detected through a double ruled grating SPEX monochromator (600 grooves/mm blazed at 1 mm) and a long-wave pass (780 nm). All the emission spectra (fluorescence and singlet oxygen luminescence) were monitored using the same absorbance (less than 0.2) in ethanol (Sigma-Aldrich) or dimethylformamide (DMF; Fisher chemical) and corrected for the lamp and photomultiplier. Rose Bengal (Acros Organics) was chosen as a reference solution thanks to its high ^1^O_2_ quantum yield in ethanol (Φ_∆0_ = 0.68)^45^ or in DMF (Φ_∆0_ = 0.40)^46^. The generation of ^1^O_2_ upon light excitation of TPAs at λ_abs,max_ (487, 485, 476, 464, 445 and 430 nm in ethanol for TP2Py, TP3Py, TP2Pyo, TP3Pyo, TP2Bzim and TP3Bzim, respectively; 481, 465, 445 and 438 nm for TP2(3)Py, TP2(3)Pyo, TP2Bzim and TP3Bzim, respectively) was then determined directly, as mentioned above, and the quantum yield of ^1^O_2_ formation (Φ_Δ_) was determined according to:

Φ_Δ_ = Φ_Δ0_ x (I/I_0_) x (A_0_/A) where I, I_0_, A and A_0_ are the luminescence intensities and the absorbances of the TPA compound and the reference, respectively.

The generation of ^1^O_2_ was also determined indirectly using two chemical probes: 1,3-Diphenylisobenzofuran (DPBF; Sigma-Aldrich) and Aminophenyl fluorescein (APF; Invitrogen) which are also sensitive to the presence of O_2_^•-^ or ^•^OH, respectively. (i) DPBF^26–28^ is a chemical probe that possesses a great selectivity for ^1^O_2_ and O_2_^•-^. In the presence of ^1^O_2_ and/or O_2_^•-^, the fluorescence emission intensity of DPBF at 510 nm (excitation at 426 nm) decreases. (ii) APF^29,30^ is non-fluorescent until it reacts with ^1^O_2_ and/or ^•^OH, but upon oxidation, APF exhibits a bright green fluorescence at 520 nm (excitation at 500 nm). The experiments with DPBF or APF were performed using a 3 mL solution of each TPA in D_2_O (Sigma-Aldrich) at a concentration of 2.6 µM (the final concentration of DPBF or APF was 26 µM). Each measurement corresponds to 21 cycles of acquisition, each cycle consists of TPA excitation for 60 s, followed by monitoring the fluorescence emission intensity at 510 and 520 nm for DPBF and APF, respectively.

### *In vitro* measurements of TPA photobleaching under continuous two-photon excitation

Photobleaching measurements were performed on a on a home-built FCS system using a 80-MHz mode-locked Mai-Tai® Ti:Sapphire tunable laser and a Nikon-TE2000 inverted microscope^47^. Two-photon excitation wavelengths are reported in the Fig. 3E legend. Fluorescence emission was separated from the excitation IR light by a Chroma 700DCSPXR dichroic mirror and an additional Chroma E700SP-2p filter. The output signal was focused onto a single-photon counting avalanche photodiode (SPCM-AQR-14, PerkinElmer). Measurements of emission intensity as a function of time were carried out in a 50-μl TPA solution (5-µM in Tris-buffer) dropped on a coverslip pre-treated with dimethyldichlorosilane.

### Fluorescence imaging

HeLa cells were seeded in glass-bottom WillCo-Dish^®^ (WillCo Wells) at 6 × 10^4^ cells/ml in DMEM (GibcoBRL) 48 h before treatments. Time-lapse (one- and two-photon) and colocalization imaging were obtained using a SP2 confocal microscope (Leica MicroSystem) equipped with an oiled immersion x63 objective (NA, 1.32) and an incubation chamber (37 °C, 5% CO_2_). A continuous laser line (458 nm) was used for TPA excitation in the confocal mode (emission slit settings: 530–690 nm for TP3Bzim and TP2Bzim; 560–720 nm for TP2Py, TP3Py, TP2Pyo and TP3Pyo). The excitation source for two-photon images was a 80-MHz mode-locked Mai-Tai^®^ Ti:Sapphire tunable laser (720–920 nm, 100-fs laser pulse; Spectra Physics). The irradiance was calculated by measuring the power at the exit of the objective using a Vega power meter (Ophir). The power measurement was performed in the x-y scan mode (image size, 512 × 512 pixels; field of view, 140 × 140 µm; scanning frequency, 800-Hz). z-stack in two-photon microscopy corresponded to 8–10 slices (512×512 pixels; z-step, 1 µm). For comparison with one-photon confocal imaging, the time scale accounting for the effective illumination time in two-photon imaging experiments was adjusted according to the z-stack as previously described^16^. Images of HeLa cells treated with both TPA and endocytosis inhibitors were obtained with a SP8 confocal microscope (Leica MicroSystem) equipped with an oiled immersion x100 objective (NA, 1.4). The continuous laser line (458 nm) was used for TPA excitation (same emission slit settings as indicated above).

Colocalization experiments (+/− endocytosis inhibitors) are described in Supplementary Methods.

### Annexin V/DAPI staining and flow cytometric analysis

Non-adherent Jurkat cells were plated in 12-well plates (10^6^ cells/well) and pre-incubated with 2-µM TPA for 2 h. Cells were then washed and subjected or not to light illumination for 30 min at 5 mW.cm^−2^ (Mercury lamp: 130 W; 380–600 nm + excitation filter: 452 ± 22.5 nm; total illuminated surface: 3.8 cm^2^ (equiv. 1 well)). After light exposure, a part of cells were immediately (t = 0) treated for Annexin V/DAPI staining whereas the rest of cells were further incubated for various times (up to 8 h) in the dark at 37 °C before Annexin V/DAPI treatment. Annexin V-FITC or Annexin V-Cy5 (Invitrogen) was used for experiments in the presence of TP2Py, TP3Py, TP2Pyo, TP3Pyo (red emission) or in the presence of TP2Bzim, TP3Bzim (green emission), respectively. For Annexin V staining, cells were washed in ice-cold PBS 1×(Gibco^®^) and stained according to manufacturer’s instructions (BD Biosciences): the cell pellet was resuspended in 100-µl Annexin V-HEPES solution (Annexin V diluted in the binding buffer: 10-mM HEPES-NaOH, pH 7.4, 140-mM NaCl, 2.5-mM CaCl_2_) and incubated for 15 min in the dark at room temperature. Cells were then further incubated with 400-µl of binding buffer containing 200-nM DAPI (Invitrogen) for 10 min. Cells were counted by flow cytometry (FACSCantoII flow cytometer; Becton-Dickinson) (≈100,000 cells analysed per condition). The laser/detection channels for fluorescence were 405-B for DAPI (excitation: 405 nm/emission: 450 ± 25 nm), 488-E for TP2Bzim, TP3Bzim and Annexin V-FITC (excitation: 488 nm/emission: 530 ± 15 nm), 488-D for TP2Py, TP3Py, TP2Pyo, TP3Pyo (excitation: 488 nm/emission: 585 ± 20 nm) and 633-C for Annexin V-Cy5 (excitation: 633 nm/emission: 660 ± 10 nm).

### Flow cytometric analysis of ROS production in the cell context

HeLa cells were seeded in 12-well plates (2.10^5^ cells/well) 24 h before treatment. Cells were pre-incubated with TPAs for 2 h at 37 °C in the dark and subjected or not to light exposure for 20 min by using a Mercury lamp (130 W; 380–600 nm + excitation filter: 452 ± 22.5 nm; irradiance: 17 mW.cm^−2^). Cells were then treated with ROS detectors for 1 h: (i) 20-µM of H_2_DCF-DA^20,21^ (Sigma-Aldrich) for TP2(3)Py- and TP2(3)Pyo-treated cells or (ii) 1X of the ROS Deep Red dye (Cellular ROS Assay Kit (ab186029); Abcam)^22^ for TP2(3)Bzim-treated cells. Cells were then washed, trypsinized and centrifuged at 1,500 rpm for 5 min. The cell pellets were resuspended in DMEM supplemented with 10% FBS (fetal bovine serum) and further analysed by flow cytometry (FACSCalibur, Becton-Dickinson) by using: (i) FL1 channel (excitation: 488 nm/emission: 530 ± 15 nm) for DCF and TP2(3)Bzim detection, (ii) FL2 channel (excitation: 488 nm/emission: 585 ± 21 nm) for TP2(3)Py and TP2(3)Pyo detection and (iii) FL4 channel (excitation: 635 nm/emission: 661 ± 8 nm) for ROS Deep Red detection. Cells treated with DMSO alone (1‰) +/− light or Tert-butyl-hydroperoxide (TBHP) (Sigma-Aldrich) at 100 µM for 1 h were used as negative and positive controls, respectively.

### Cellular uptake inhibition: Fluorescence microscopy

Energy dependence experiments were performed with HeLa cells, first pre-incubated for 5 min at 4 °C before incubation with 2-µM TPA in cold medium for 60 min at 4 °C. Cells were then washed with ice cold PBS buffer (Gibco^®^) 3 times for 5 min to remove extracellular TPA. For the control, cells were incubated with 2-µM TPA for 60 min at 37 °C, and washed with PBS buffer 3 times for 5 min. For endocytosis inhibition experiments, HeLa cells were pre-treated with endocytosis inhibitors (30-µM chlorpromazine, 80-µM dynasore, 5-µM cytochalasin D or 100-µM-genistein) or with the solvent used to dissolve the inhibitor for 30 min at 37 °C before addition of 2-µM TPA and further incubation for 30, 60 or 90 min at 37 °C. All inhibitors were purchased from Sigma-Aldrich. The quantitative analysis of TPA fluorescence intensity in cells treated or not with endocytosis inhibitors (3 independent experiments for each condition; ≈50 cells analyzed per experiment) is explained in Supplementary Methods.

### Cellular uptake inhibition: Flow cytometry

HeLa cells were plated in 12-well plates (10^5^ cells/well) 24 h before experiments. Cells were pre-treated with endocytosis inhibitors (see previous section) or with the solvent used to dissolve the inhibitor for 30 min before addition of 2-µM TPA and further incubation for 90 min at 37 °C (TPA-incubation time of 30 min was also performed in the case of genistein). Cells were washed with PBS buffer, trypsinized and transferred in FACS tubes. After centrifugation (1,500 rpm; 5 min), cell pellets were resuspended in 500-µl of fresh medium before measurement. The measurement was done by a FACSCalibur flow cytometer (Becton-Dickinson) using the 488 nm laser line for TPA excitation. Emission was measured at 585 ± 21 nm (FL2 channel) for TP2Py and 530 ± 15 nm (FL1 channel) for TP2(3)Bzim. The analysis of results (3 independent experiments for each condition; ≈30,000 cells analyzed per experiment) is explained in Supplementary Information (Supplementary Fig. S9).

## Supplementary information

Supplementary information.
