# Supplementary material for: Interplay between Cellular Uptake, Intracellular Localization and the Cell Death Mechanism in Triphenylamine-Mediated Photoinduced Cell Death
Source: Sci Rep. 2020 Apr 23;10:6881. doi: 10.1038/s41598-020-63991-9 (PMC7181850; doi:10.1038/s41598-020-63991-9)
Supplement: Supplementary file 1 — Supplementary information. [file 41598_2020_63991_MOESM1_ESM.pdf]

Supplementary Information

Interplay between Cellular Uptake, Intracellular Localization and the Cell Death Mechanism in Triphenylamine-Mediated Photoinduced Cell Death.

Rahima Chennoufi[1], Ngoc-Duong Trinh[1], Françoise Simon[1], Guillaume Bordeau[2,†], Delphine Naud-Martin[2], Albert Moussaron[3], Bertrand Cinquin[1], Houcine Bougherara[4], Béatrice Rambaud[1], Patrick Tauc[1], Céline Frochot[3], Marie-Paule Teulade-Fichou[2,*], Florence Mahuteau-Betzer[2,*] & and Eric Deprez[1,*]

[1]Laboratory of Biology and Applied Pharmacology (LBPA), CNRS UMR8113, IDA FR3242, ENS Paris-Saclay, Université Paris-Saclay, F-91190 Gif-sur-Yvette, France.

[2]UMR9187, CNRS, INSERM, Institut Curie, PSL Research University, Université Paris-Saclay, F-91405 Orsay, France.

[3]LRGP, UMR7274 CNRS-Université de Lorraine, F-54000 Nancy, France.

[4]Institut Cochin, INSERM U1016-CNRS UMR8104-Université Paris Descartes, Sorbonne Paris Cité, F-75014 Paris, France; present address: Institut de Recherches Servier SA, F-78290 Croissy-sur-Seine, France.

[†]Present address: Laboratoire des IMRCP, Université de Toulouse, CNRS UMR5623, Université Toulouse-III - Paul Sabatier, F-31400 Toulouse, France.

*Correspondence and requests for materials should be addressed to E.D. (email: deprez@lbpa.ens-cachan.fr), F.M.-B. (florence.mahuteau@curie.fr) or M.-P.T.-F. (mp.teulade-fichou@curie.fr).

Table of contents:

- Table S1……..………………………………………..……..page 2
- Figure S1…………………………………..……………………page 3
- Figure S2..……………………..………..……………………page 4
- Figure S3..……………………..………..……………………page 5
- Figure S4……………………………………………………….page 6
- Figure S5…………………………………..……………………page 7
- Figure S6……..……………………………………………..…….page 8
- Figure S7..page 9
- Figure S8..page 10
- Figure S9..page 14
- Supplementary Methods...page 15
- Supplementary References...page 17

Table S1. Absorption, 1- and 2-photon fluorescence properties of TPA compounds.

		$\lambda_{abs/exc\ (1\text{-}h\nu)}$ (nm)[a]	ε ($M^{-1}.cm^{-1}$)[a]	$\lambda_{exc\ (2\text{-}h\nu)}$ (nm)[b]	σ^2 (GM)[b]	λ_{em} (nm)[c]	ϕ_F[c]	σ^2 x ϕ_F (GM)[c]	Φ_Δ[d]
TP2Py[g]	- DNA[e]	473	39,900	840	n.d.	633	n.d.	0.3	≤ 0.01
	+ DNA[e]	506	31,400	860 (840)	325 (260)	637	0.08	26 (18)	
TP3Py	- DNA	477	59,000	810	2.5	656	n.d.	n.d.	≤ 0.01
	+ DNA	501	51,000	830 (840)	500 (460)	641	0.01	5 (4.6)	
TP2Bzim	- DNA	439	42,500	800	1.6	607	n.d.	n.d.	≤ 0.01
	+ DNA	476	44,400	830 (840)	495 (1080)	576	0.54	267 (583)	
TP3Bzim[g]	- DNA	433	68,900	740	n.d.	620	n.d.	2	≤ 0.01
	+ DNA	457	62,400	760[f]<br>840[f]	764<br>250	572	0.34	258<br>85	
TP2Pyo	- DNA	460	47,700	830	1.6	637	n.d.	n.d.	≤ 0.01
	+ DNA	494	41,500	850	365	610	0.17	62	
TP3Pyo	- DNA	456	63,900	880	2.3	638	n.d.	n.d.	≤ 0.01
	+ DNA	485	65,100	830	388	611	0.08	31	

[a]Maximum 1-photon absorption/excitation (1-hν) wavelength; ε: corresponding molar extinction coefficient.
[b]Maximum 2-photon excitation (2-hν) wavelength; σ^2: corresponding 2-photon absorption cross-section.
[c]Maximum emission wavelentgh; ϕ_F: fluorescence quantum yield (ϕ_F values were obtained according to Dumat *et al.* 2013[1]); σ^2 x ϕ_F: 2-photon brightness.
[d]Φ_Δ: $^1O_2$ quantum yields in ethanol or DMF (similar results were obtained, regardless of the solvent used). Φ_Δ values were determined using Rose Bengal as a reference: $\Phi_{\Delta 0}$ = 0.68 and 0.4 in ethanol and DMF, respectively (see also Fig. S4A).
[e]Absorption and fluorescence measurements were performed with 5 µM TPA in 10 mM Tris pH 7.4, 100 mM KCl, in the absence or presence of 10 µM double-stranded 21-mer DNA according to Chennoufi *et al.* 2016[2] (the DNA sequence is available in this reference). Values in parentheses are reported values obtained using a self complementary 14-mer DNA (DrewAT)[1].
[f]Two excitation maxima.
[g]Except Φ_Δ, all data relative toTP2Py and TP3Bzim are from reference[2].

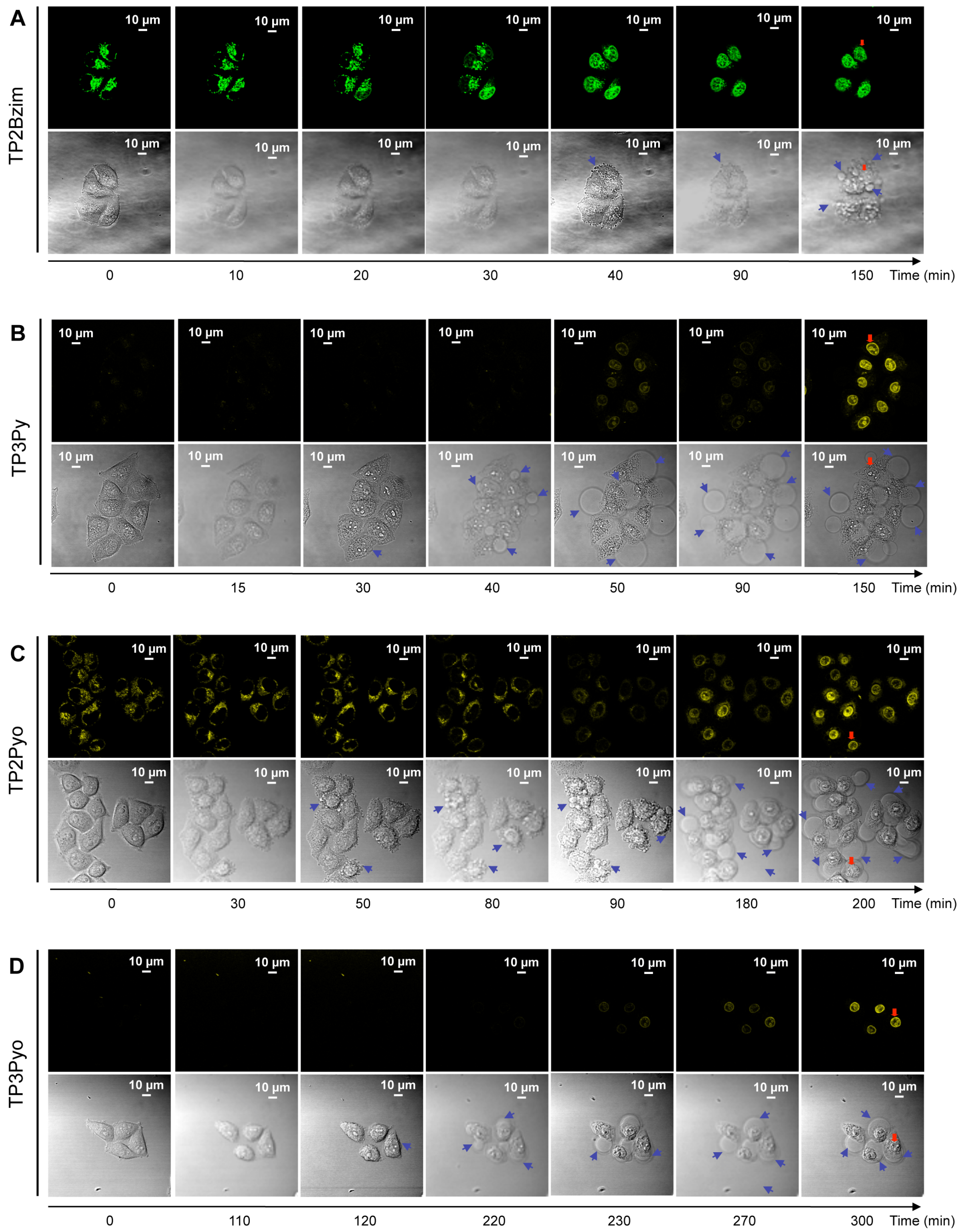

Figure S1. Time-lapse confocal fluorescence microscopy imaging of intracellular events caused by prolonged visible light irradiation (458 nm) of TPAs. All experimental conditions are provided in the Fig. 2 legend. Living HeLa cells were pre-treated with 2-µM TPA (**A**: TP2Bzim; **B**: TP3Py; **C**: TP2Pyo; **D**: TP3Pyo) for 2h at 37°C before continuous irradiation (starting at t = 0). Fluorescence images show nuclear translocation of TPAs whereas the concomitant appearance of plasma membrane blebs can be easily observed in the DIC – differential interference contrast – transmission mode (indicated by blue arrows).

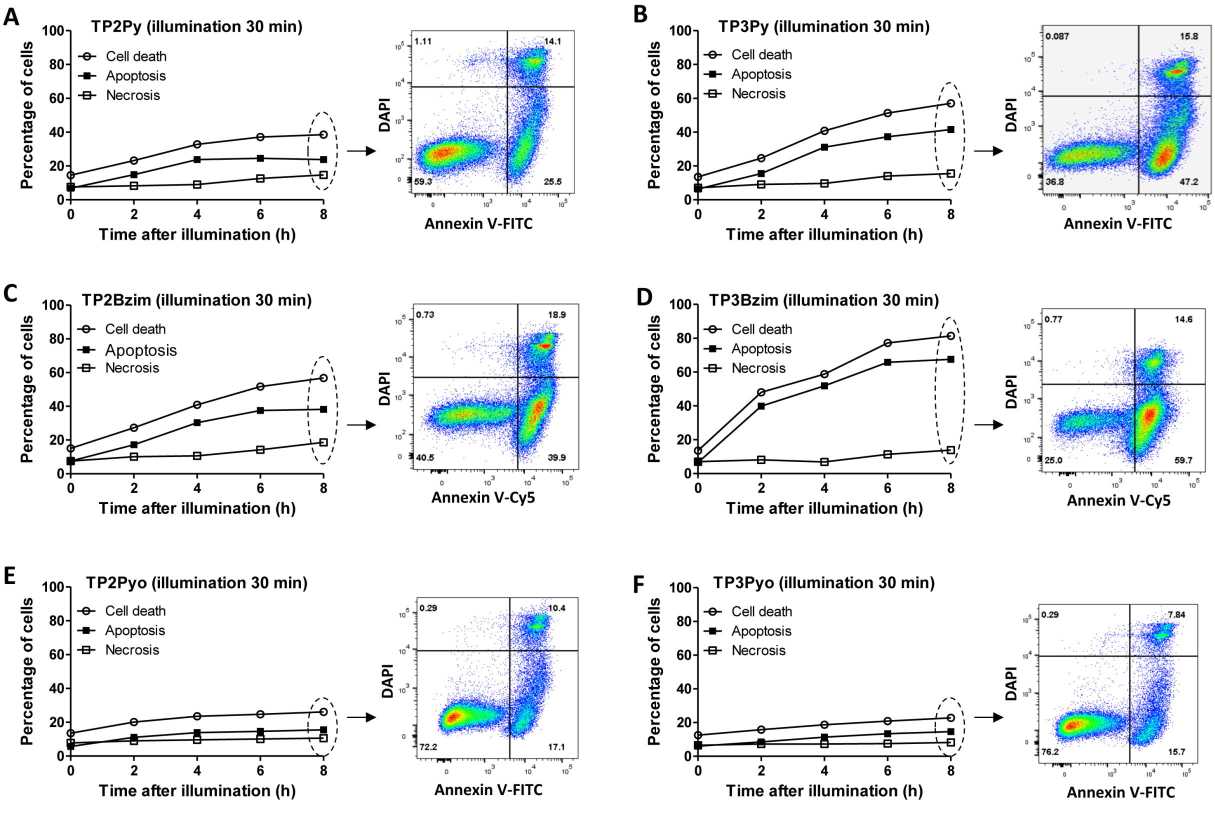

Figure S2. Apoptotic and necrotic cell subpopulations as a function of time after TPA photoactivation. Living Jurkat cells were treated with 2-µM TP2Py (**A**), TP3Py (**B**), TP2Bzim (**C**), TP3Bzim (**D**), TP2Pyo (**E**) or TP3Pyo (**F**) and subjected to light illumination (5 mW.cm^{-2}) for 30 min (Mercury lamp: 130W; 380-600 nm + excitation filter: 452±22.5 nm). After light exposure, a part of cells were immediately (t = 0) treated for Annexin V/DAPI staining whereas the rest of cells were further incubated for various times (up to 8h) in the dark at 37°C before Annexin V/DAPI treatment and flow cytometric analysis (see Methods for experimental details). Black squares: apoptotic cells (Annexin V$^+$/DAPI$^-$); white squares: necrotic cells (Annexin V$^+$/DAPI$^+$); white circles: total number of dead cells (Annexin V$^+$, DAPI$^{+/-}$). The plots show representative values of 3 independent experiments. Right: examples of 2-D dot plots for t = 8h. Negative and positive control populations are shown in Fig. S3.

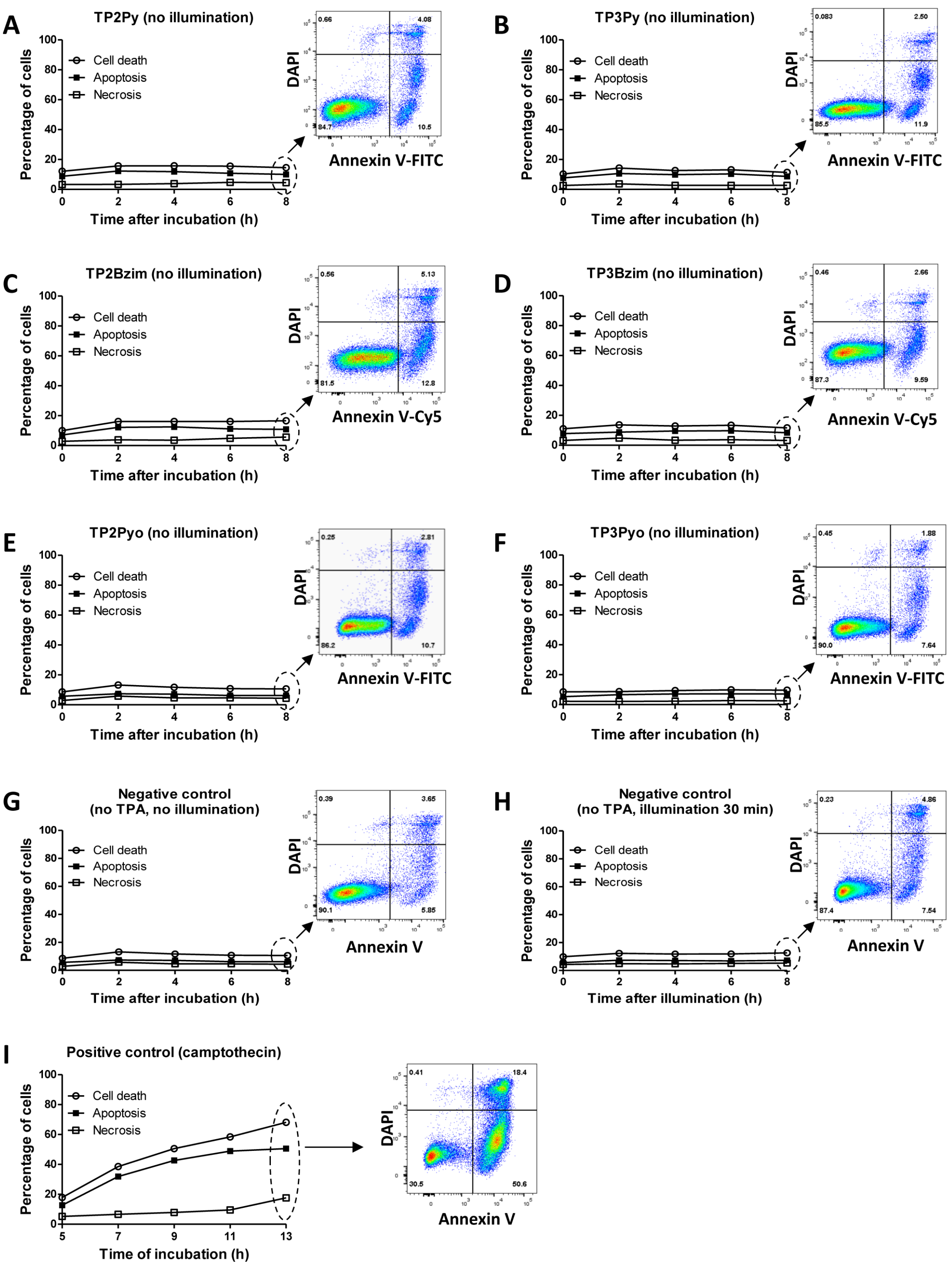

Figure S3. Negative and positive controls of flow cytometry experiments with Annexin V/DAPI double staining. (**A-F**) Control populations of TPA-treated cells without light exposure. Living Jurkat cells were treated with 2-µM TPA (TP2Py (**A**), TP3Py (**B**), TP2Bzim (**C**), TP3Bzim (**D**), TP2Pyo (**E**) or TP3Pyo (**F**)) as explained in the Fig. S2 legend, except that TPA-treated cells were not subjected to light illumination. (**G-H**) Control populations of untreated cells without (**G**) or with light exposure (**H**). (**I**) Positive control populations of camptothecin-treated cells. Jurkat cells were treated with 10-µM camptothecin for 5-to-13h in the dark at 37°C and analysed by flow cytometry. Black squares: apoptotic cells (Annexin V^+/$DAPI^-$); white squares: necrotic cells (Annexin V^+/$DAPI^+$); white circles: total number of dead cells (Annexin V^+, $DAPI^{+/-}$). The plots show representative values of 3 independent experiments. Right: examples of 2-D dot plots for t = 8h (13h for camptothecin).

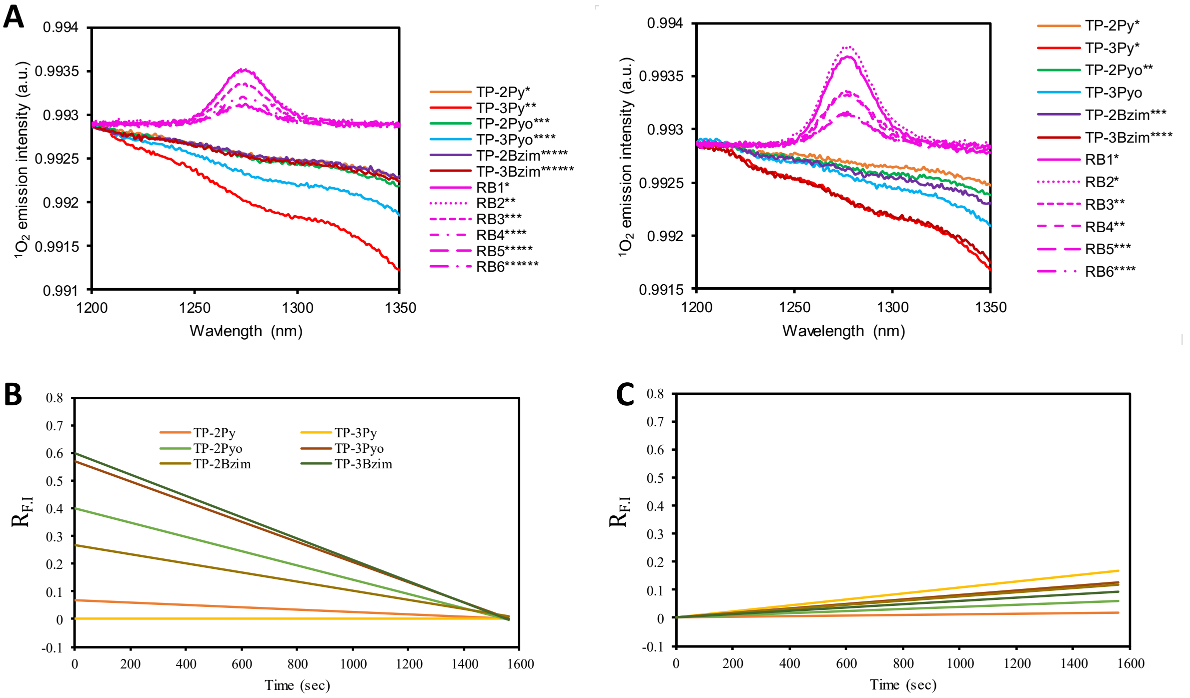

Figure S4. *In vitro* measurements of ROS production upon TPA photoactivation. (**A**) $^1O_2$ luminescence spectra upon TPA excitation. All emission spectra were measured in ethanol (left) or DMF (right) between 1200 and 1350 nm. Each TPA was excited at its optimal $\lambda_{abs,max}$, as indicated in the "Methods" section. A reference spectrum, using Rose Bengal (RB), was monitored for each excitation condition (RB excited at the corresponding $\lambda_{abs,max}$): *487 nm, **485 nm, ***476 nm, ****464 nm, *****445 nm, ******430 nm in ethanol and *481 nm, **465 nm, ***445 nm and ****438 nm in DMF. The quantum yields of $^1O_2$ formation (Φ_Δ) are reported in Table S1. (**B-C**) Variation of the fluorescence emission intensity of the DPBF (**B**) or the APF (**C**) probe in D_2O upon TPA excitation. DPBF and APF are ROS detectors (selective for $O_2^{\bullet-}$/$^1O_2$ and $^{\bullet}OH$/$^1O_2$, respectively) that display decrease and increase in the emission intensity at 510 (excitation: 426 nm) and 520 nm (excitation: 500 nm) in the presence of ROS, respectively (see Methods for experimental details). For each probe, the relative variation of the fluorescence intensity is represented and expressed by the ratio $R_{F.I} = \Delta F.I / F.I(t_{ref})$ with $\Delta F.I = F.I(t) - F.I.(t_{ref})$.

F.I(t) corresponds to the fluorescence intensity at time t; $F.I(t_{ref})$ corresponds to $F.I(t_f)$ or $F.I(t_0)$ for DPBF and APF, respectively, where $F.I(t_f)$ and $F.I(t_0)$ are the fluorescence intensities at final time and time 0, respectively.

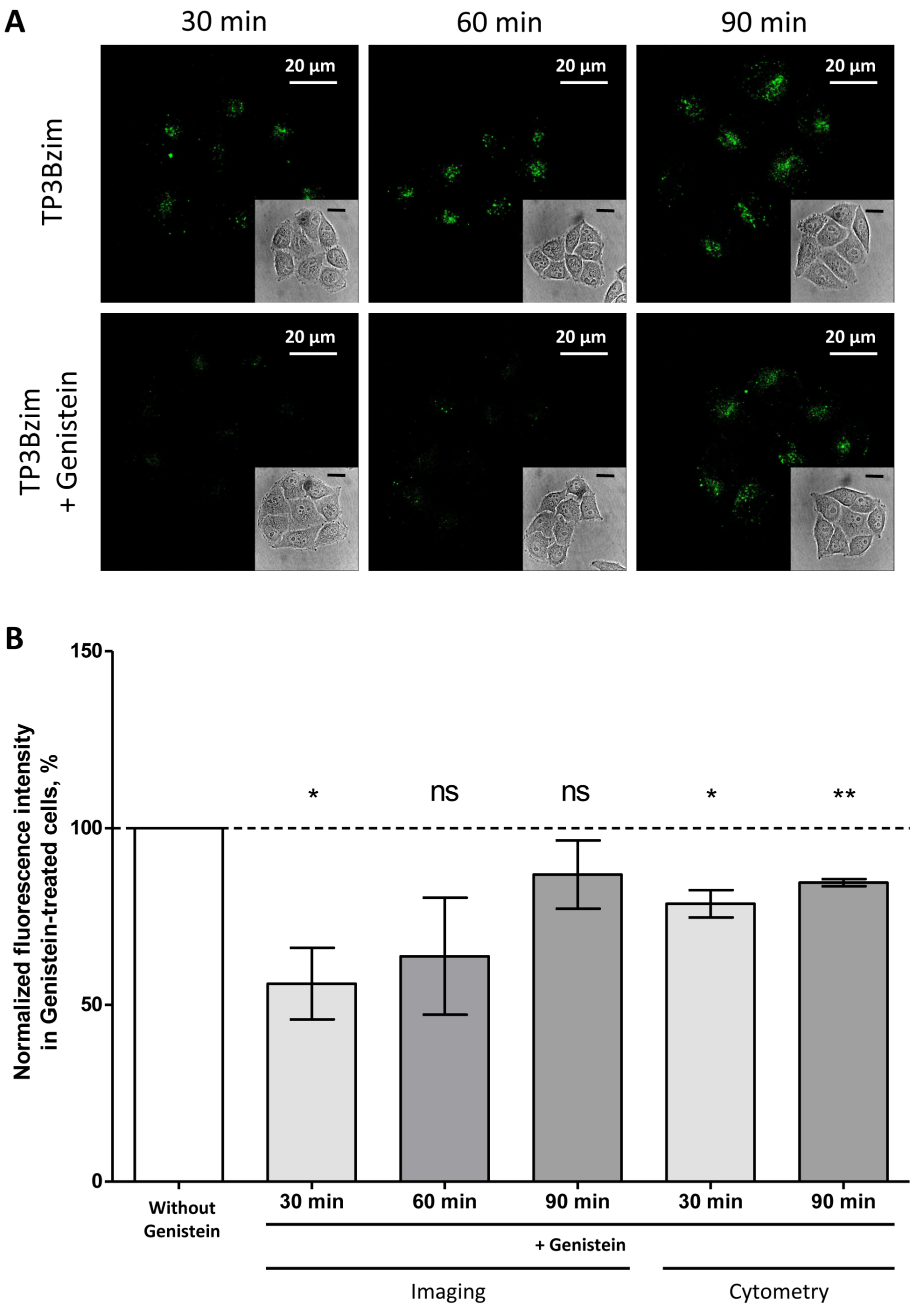

Figure S5. Effect of genistein on TP3Bzim cellular uptake as a function of TP3Bzim-incubation time. HeLa cells were pre-treated with genistein for 30 min at 37°C before addition of 2-µM TP3Bzim and further incubation for 30, 60 or 90 min at 37°C. Cells were then analysed by fluorescence imaging or flow cytometry (see Methods for experimental details). (**A**) Fluorescence images for each TP3Bzim-incubation time with corresponding DIC images (insets): 30 min (left), 60 min (middle) and 90 min (right). Top: without genistein. Bottom: +100-µM-genistein. (**B**) Quantitative analysis of TP3Bzim fluorescence intensity in genistein-treated cells corresponding to fluorescence imaging (left) and flow cytometry experiments (right) (normalized for each incubation time by the intensity level obtained in the absence of inhibitor). The bar graphs show mean±SD values from 3 independent experiments, *$p<0.05$, **$p<0.01$, ns: not significant (statistical significance was determined using the paired t-test and two-way ANOVA (GraphPad Prism 5)). Altogether, the results show that the genistein-mediated inhibition of TP3Bzim uptake is dependent on the TP3Bzim-incubation time with higher inhibition efficiency obtained for shorter incubation times.

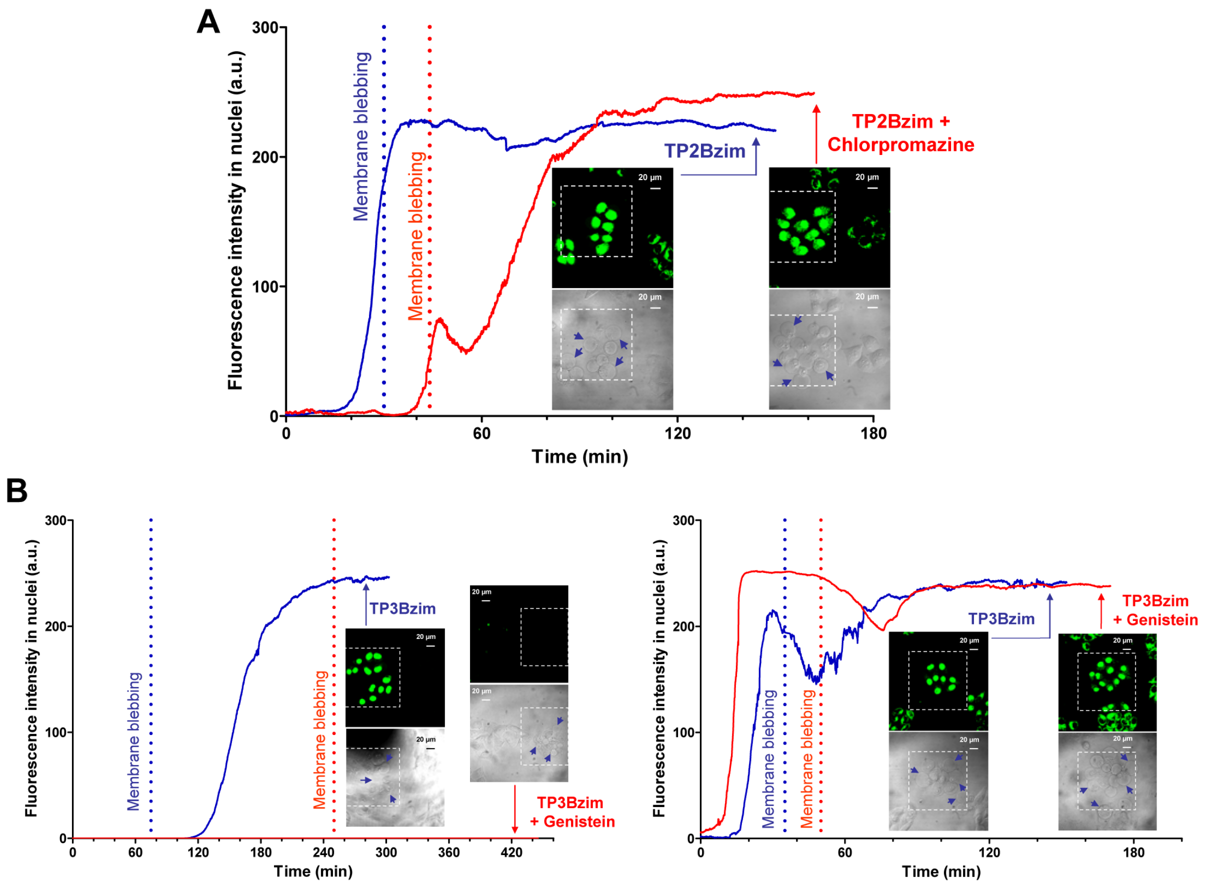

Figure S6. Kinetics of nuclear accumulation of TPAs in HeLa cells treated (red) or not (blue) with endocytosis inhibitors and further subjected to light illumination. (**A**) TP2Bzim+chlorpromazine. (**B**) TP3Bzim+genistein. HeLa cells were pre-treated with endocytosis inhibitors for 30 min at 37°C before addition of 2-µM TPA and further incubation. TPA-incubation time was 90 min in the case of TP2Bzim (A), 30 min (B, left) or 90 min (B, right) in the case of TP3Bzim. After TPA-incubation, cells were continuously irradiated (conditions of light illumination are similar to those described in the Fig. 2 legend). For each condition, the dashed line indicates the appearance of first membrane blebs; fluorescence/DIC images are also shown at the end of the process. These images show cells characterized by both a nuclear translocation of the TPA emission signal (fluorescence images) and membrane blebbing (blue arrows in DIC images) that specifically occur in the irradiated area (delineated by dashed lines).

As shown in panel A, chlorpromazin that efficiently inhibits the cellular uptake of TP2Bzim (see Fig. 7B-D) also delays both TPA nuclear translocation ($\Delta_{translocation}$ = +20-40 min) and the appearance of membrane blebs ($\Delta_{blebbing}$ ≈ +15 min), in a similar manner to that observed for TP2Py in Fig. 7E ($\Delta_{translocation}$ ≈ +45 min and $\Delta_{blebbing}$ ≈ +40 min). Regarding the “TP3Bzim+genistein” condition (panel B), the genistein effects on nuclear translocation and membrane blebbing are strongly dependent on the TPA-incubation time with more significant effects observed for an incubation time of 30 min (left: no detectable fluorescence signal in nuclei and $\Delta_{blebbing}$ ≈ +175 min) than for an incubation time of 90 min (right: $\Delta_{translocation}$ ≈ -8 min and $\Delta_{blebbing}$ ≈ +14 min) in accordance with the influence of the TP3Bzim-incubation time on the TP3Bzim cellular uptake (Fig. S5).

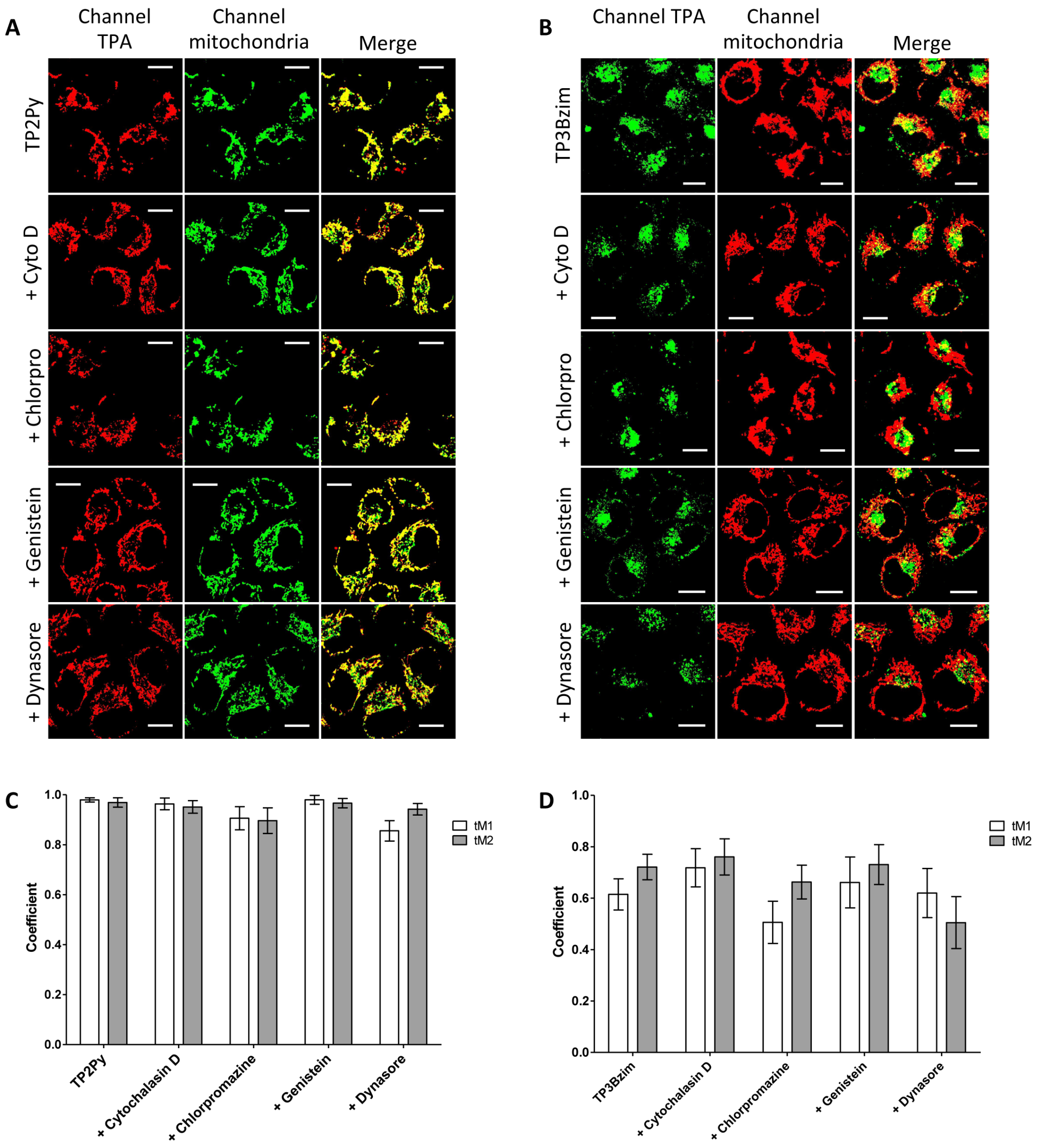

Figure S7. Colocalization between TPAs and mitochondria in the presence of endocytosis inhibitors. Colocalization experiments were performed with living HeLa cells, treated with 2-µM TP2Py (**A**) or TP3Bzim (**B**) in the absence (line 1) or presence of endocytosis inhibitors (lines 2-5), and further observed using confocal microscopy. Left, imaging channels of TPAs. Middle, imaging channels of organelle trackers. Right, merged images (scale bar indicates 10-µm). Yellow-to-orange areas indicate TPA/mitochondria colocalization. Mitochondria staining was performed using MitoTracker® Green FM (λ_{exc} = 488 nm / emission slit: 500-550 nm) for TP2Py (λ_{ex} = 458 nm / emission slit: 560-720 nm) or MitoTracker® Red FM (λ_{exc} = 633 nm / emission slit: 650-700 nm) for TP3Bzim (λ_{ex} = 458 nm / emission slit: 530-690 nm). The quantitative evaluation of colocalization was performed by using the Manders' Colocalization Coefficients (MCC): tM1 and tM2 (see Supplementary Methods). tM1/tM2 values in the absence or presence of endocytosis inhibitors are shown for TP2Py (**C**) and TP3Bzim (**D**). The bar graphs in panels C-D show mean±SD values from 35 measurements for each condition.

The results show that endocytosis inhibitors do not significantly influence the mitochondrial localization of TP2Py or TP3Bzim, even when these inhibitors strongly affect TPA cellular uptake (*e.g.* chlorpromazine for TP2Py, dynasore for both TP2Py and TP3Bzim). The highest tM1 and tM2 values obtained for TP2Py (0.90-0.98 compared to 0.5-0.76 for TP3Bzim) are in accordance with results in Fig. 4 showing that the degree of colocalization with mitochondria is consistently higher for TP2 compounds than for TP3 compounds.

A

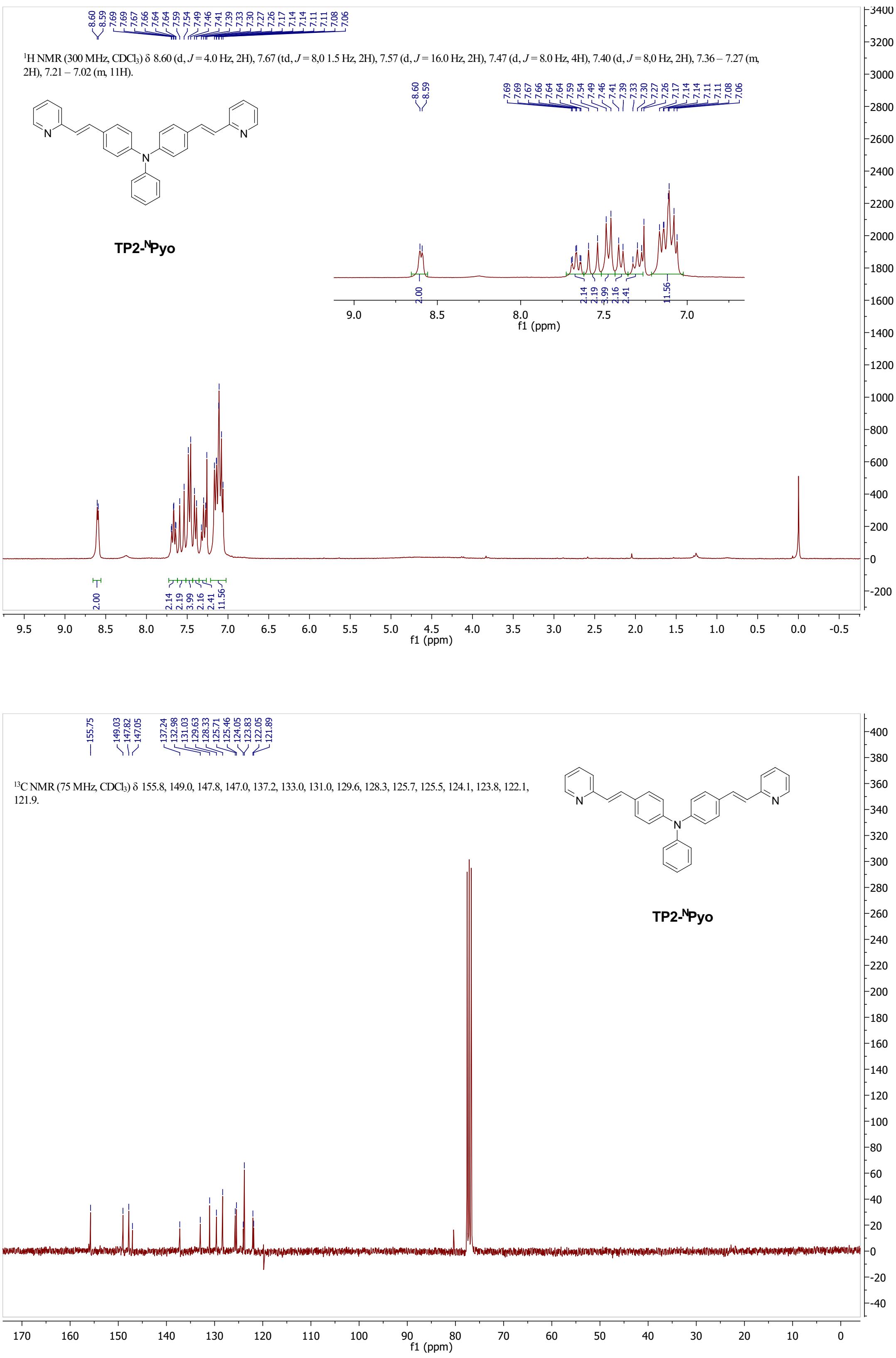

Figure S8A. NMR spectra of TP2-^{N}Pyo (top: ^{1}H; bottom: ^{13}C).

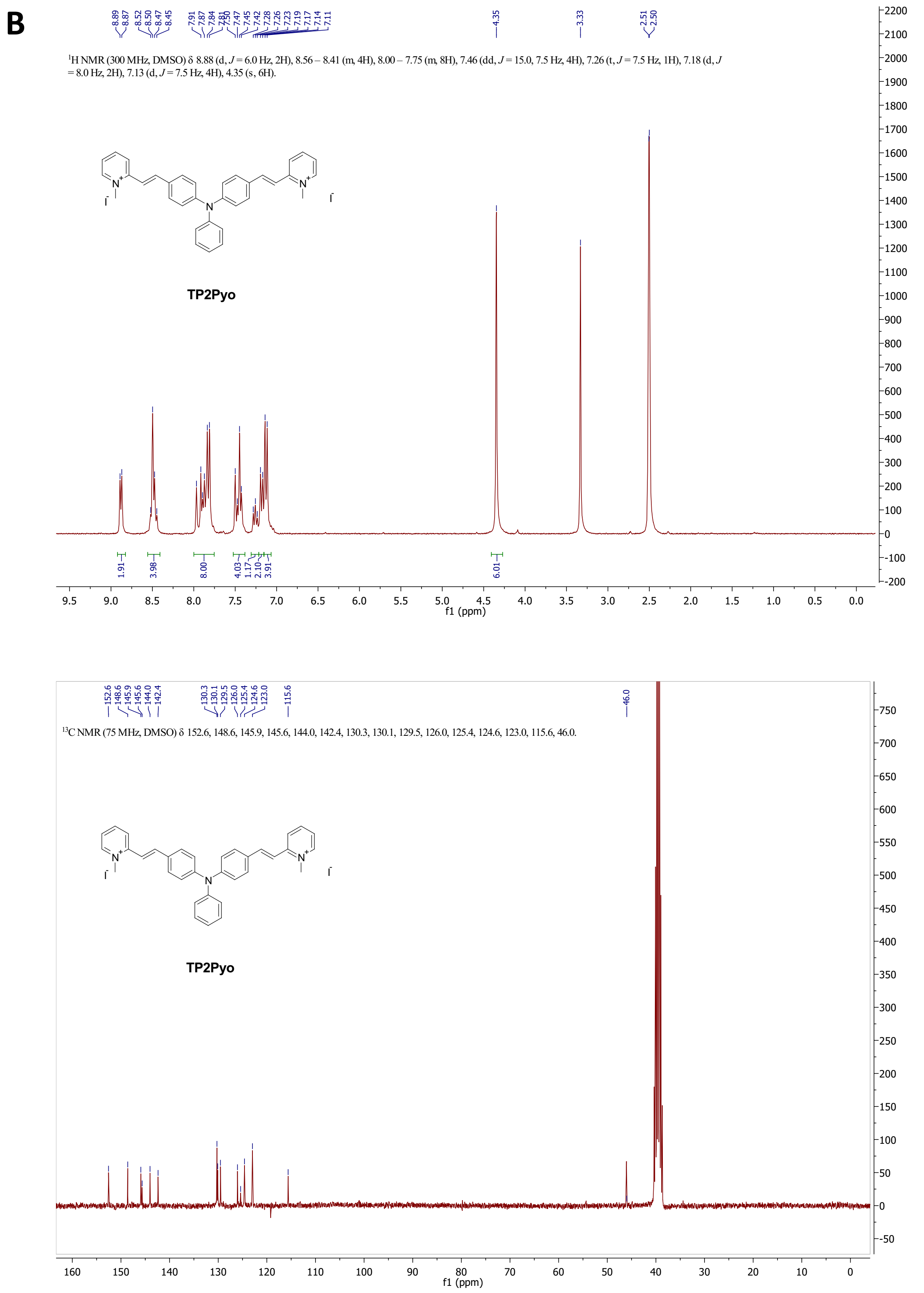

Figure S8B. NMR spectra of TP2-Pyo (top: ^{1}H; bottom: ^{13}C).

C

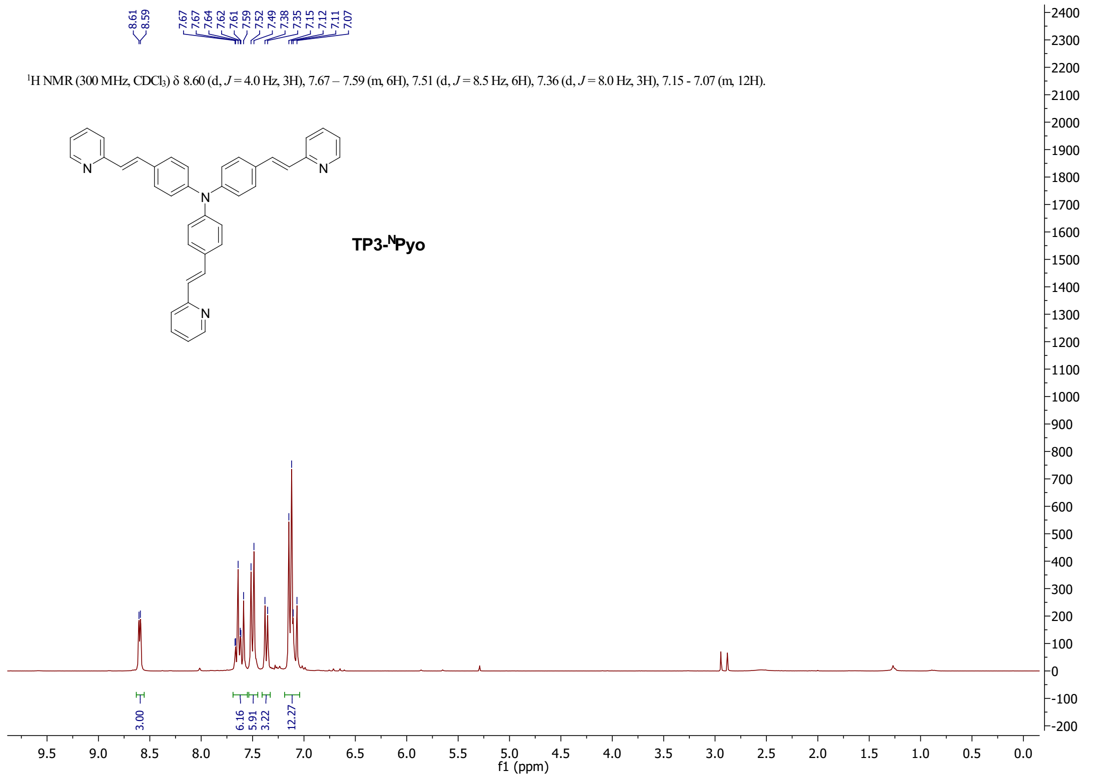

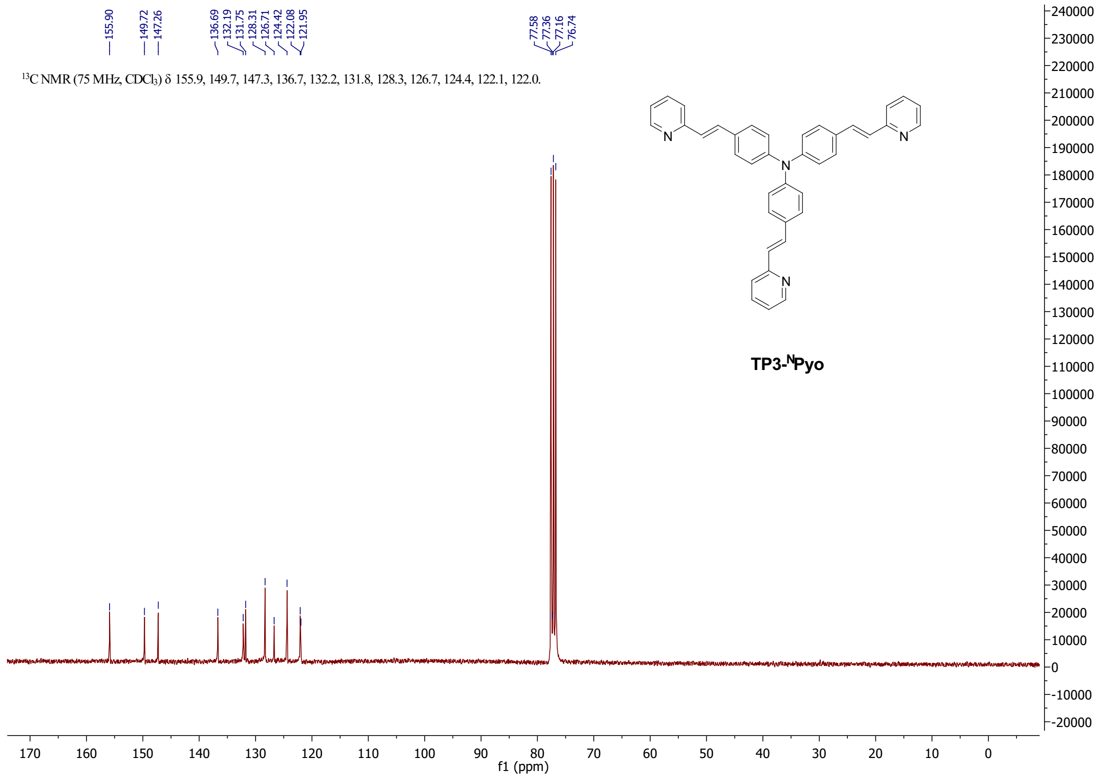

Figure S8C. NMR spectra of TP3-^{N}Pyo (top: ^{1}H; bottom: ^{13}C).

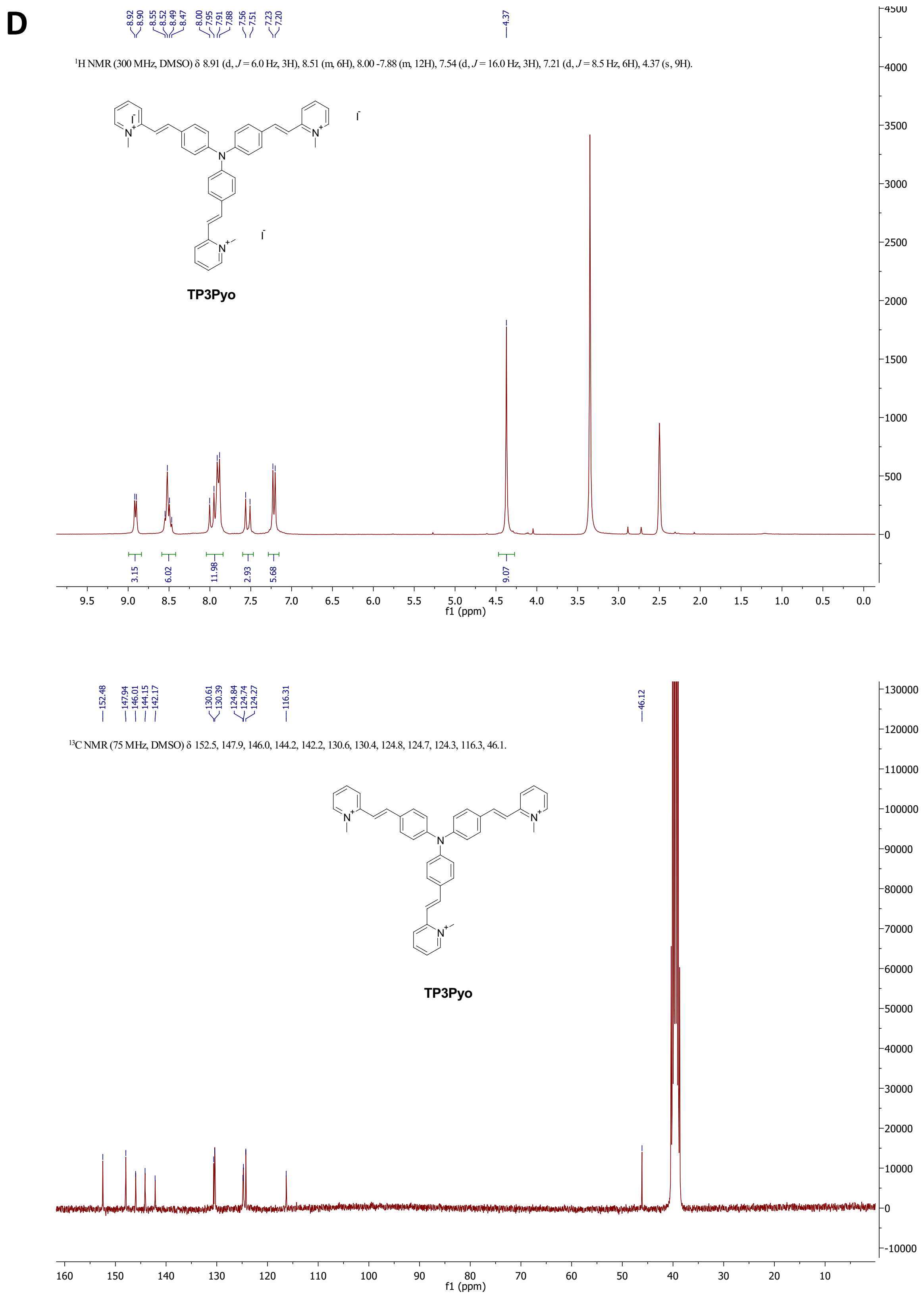

Figure S8D. NMR spectra of TP3-Pyo (top: ^{1}H; bottom: ^{13}C).

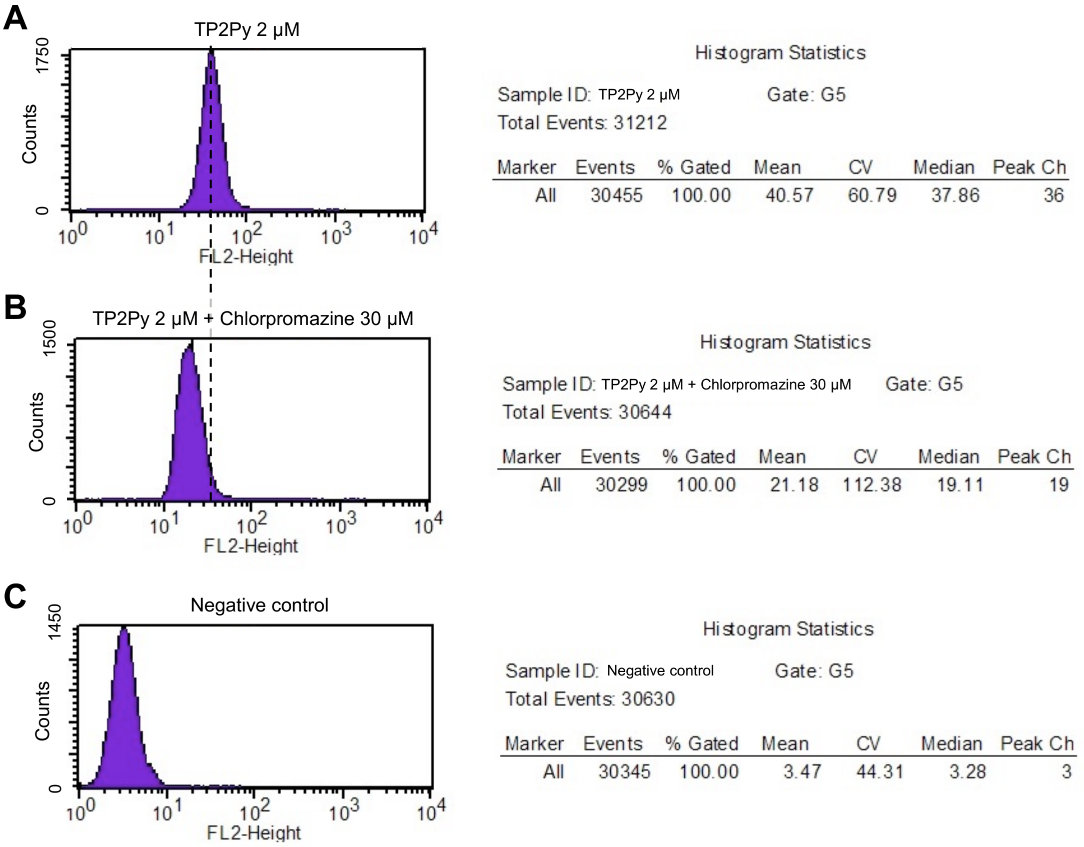

Figure S9. Example of flow cytometry analysis of TPA cellular uptake inhibition by endocytosis inhibitors. HeLa cells were treated with TP2Py in the absence (**A**: TPA+,Inhi-) or presence of chlorpromazin (**B**: TPA+,Inhi+) (see Methods for details about the experimental procedure). (**C**) no TP2Py, no chlorpromazin (TPA-,Inhi-). The uptake percentage was calculated according to the median fluorescence intensity (MFI): % (uptake) = $[MFI^{TPA+,Inhi+} - MFI^{TPA-,Inhi-}] / [MFI^{TPA+,Inhi-} - MFI^{TPA-,Inhi-}]$ x100.

Supplementary Methods

Synthesis of TP2(3)Pyo.

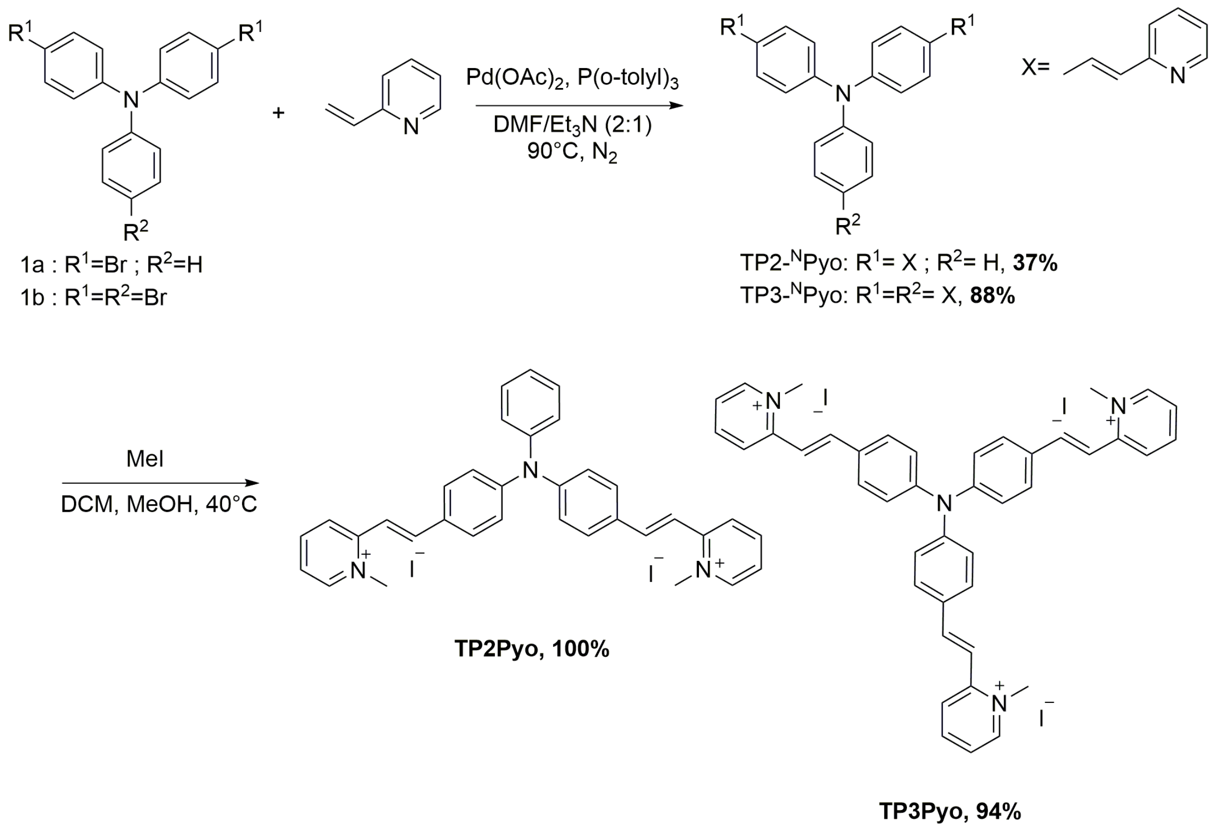

General statement:

All solvents were purchased from Carlo Erba at reagent grade. Triethylamine (Et_3N) was distilled over calcium hydride (CaH_2) and stored over barium oxide (BaO). Starting materials were purchased from Aldrich, Alfa Aesar and Acros without further purification. All reactions were carried out under argon atmosphere. Reactions were monitored by thin-layer chromatography on Macherey Nagel AlugramXtraSiL G/UV254 and flash chromatography was carried out with Macherey Nagel silica gel (40-63 µm) or on CombiFlash Companion from Teledyne Isco equipped with packed silica cartridges from Macherey Nagel.Yields refer to chromatographically and spectroscopically pure compounds.^{1}H and ^{13}C NMR spectra were recorded at 300 MHz and 75 MHz respectively on a Bruker AC-300 spectrometer at ambient temperature using an internal deuterium lock. DMSO-d_6 and $CDCl_3$ were purchased from Eurisotop. Proton chemical shifts are reported in ppm (δ) with the solvent reference as the internal standard (DMSO-d_6, δ 2.50 ppm; $CDCl_3$, δ 7.26 ppm). Data are reported as it follows: chemical shift (multiplicity [singlet (s), doublet (d), triplet (t) and multiplet (m)], coupling constants [Hz], integration). LC-MS spectra (ESI in the positive ion mode) were performed with a Waters ZQ instrument (source voltage 50-75 kV). Electrospray ionization high resolution mass spectrometry (HRMS) was performed of the Small Molecule Mass Spectrometry platform of IMAGIF (Centre de Recherche de Gif - www.imagif.cnrs.fr).

Synthesis of N,N-Bis[4-(2-pyridylethenyl)phenyl]aniline (TP2-^{N}Pyo):

In a dry and degassed triethylamine/DMF (2:1, v/v) mixture, palladium acetate (10 mg, 0.04 mmol, 0.07 equiv.) and tris-*o*-tolylphosphine (27 mg, 0.09 mmol, 0.15 equiv.) were introduced and stirred for 15 min. Mixture was degassed then 4-bromo-N-(4-bromophenyl)-N-phenylaniline 1a (240 mg, 0.6 mmol, 1.0 equiv.) and 2-vinylpyridine (190 mg, 1.8 mmol, 3 equiv.) were added. The mixture was stirred at 85-90°C overnight under nitrogen. The resulting mixture was cooled to room temperature and diluted

with dichloromethane, washed with water and saturated aqueous solution of $NaHCO_3$ and dried over $MgSO_4$. After concentration to dryness, the crude residue was purified on column chromatography (gradient from CH_2Cl_2/EtOH 100/0 to 95/5). Fractions were collected and after concentration, the residue was precipitated with a mixture of Et_2O/n-pentane and filtered to afford the title compound as a red solid in 37 % yield (100 mg, 0.22 mmol). ^1H NMR (300 MHz, $CDCl_3$) δ 8.60 (d, J = 4.0 Hz, 2H), 7.67 (td, J = 8,0 1.5 Hz, 2H), 7.57 (d, J = 16.0 Hz, 2H), 7.47 (d, J = 8.0 Hz, 4H), 7.40 (d, J = 8,0 Hz, 2H), 7.36 – 7.27 (m, 2H), 7.21 – 7.02 (m, 11H). ^{13}C NMR (75 MHz, $CDCl_3$) δ 155.8, 149.0, 147.8, 147.0, 137.2, 133.0, 131.0, 129.6, 128.3, 125.7, 125.5, 124.1, 123.8, 122.1, 121.9. LR-MS (ESI-MS): m/z 452.0 $[M+H]^+$. HRMS (ESI+): m/z calculated for $C_{32}H_{26}N_3$: 452.2127; found: 452.2124.

Synthesis of Tris[4-(2-pyridylethenyl)phenyl]amine (TP3-^{N}Pyo):
This compound was prepared as described above, except that $Pd(OAc)_2$ (10 mg, 0.04 mmol, 0.07 equiv.), P(*o*-Tol)$_3$ (27 mg, 0.09 mmol, 0.15 equiv.), tris-(4-bromophenyl)amine 1b (400 mg, 0.64 mmol, 1 equiv.) and 2-vinylpyridine (270 mg, 2.56 mmol, 4 equiv.) were used. The title compound was obtained as a red solid in 88 % yield (315 mg, 0.56 mmol). ^1H NMR (300 MHz, $CDCl_3$) δ 8.60 (d, J = 4.0 Hz, 3H), 7.67 – 7.59 (m, 6H), 7.51 (d, J = 8.5 Hz, 6H), 7.36 (d, J = 8.0 Hz, 3H), 7.15 - 7.07 (m, 12H). ^{13}C NMR (75 MHz, $CDCl_3$) δ 155.9, 149.7, 147.3, 136.7, 132.2, 131.8, 128.3, 126.7, 124.4, 122.1, 122.0. LR-MS (ESI-MS): m/z 555.0 $[M+H]^+$. HRMS (ESI+): m/z calculated for $C_{39}H_{31}N_4$: 555.2549; found: 555.2550.

Synthesis of N,N-Bis[4-(N-methyl-2-pyridinioethenyl)phenyl]-N-phenylamine bisiodide (TP2Pyo):
A large excess of methyl iodide (1 mL) was added to a solution of compound TP2-^{N}Pyo (20 mg, 0.04 mmol) in methanol/dichloromethane mixture. The solution was stirred at reflux in the dark overnight. The reaction mixture was concentrated to dryness. The crude residue was dissolved in a minimal amount of methanol and precipitated by addition of diethyl ether, affording the desired compound as a dark red solid (32 mg, 0.04 mmol) in quantitative yield. mp >260°C. ^1H NMR (300 MHz, DMSO-d_6) δ 8.88 (d, J = 6.0 Hz, 2H), 8.56 – 8.41 (m, 4H), 8.00 – 7.75 (m, 8H), 7.46 (dd, J = 15.0, 7.5 Hz, 4H), 7.26 (t, J = 7.5 Hz, 1H), 7.18 (d, J = 8.0 Hz, 2H), 7.13 (d, J = 7.5 Hz, 4H), 4.35 (s, 6H). ^{13}C NMR (75 MHz, DMSO-d_6) δ 152.5, 147.9, 146.0, 144.2, 142.2, 130.6, 130.4, 124.8, 124.7, 124.3, 116.3, 46.1. LR-MS (ESI-MS): m/z 240.1 $[M-2I^-]^{2+}$. HRMS (ESI+): m/z calculated for $[C_{34}H_{31}N_3]^{2+}$: 240.6259; found: 240.6257.

Synthesis of Tris[4-(N-methyl-2-pyridinioethenyl)phenyl]amine trisiodide (TP3Pyo):
This compound was prepared as described above, except that compound TP3-^{N}Pyo (50 mg, 0.09 mmol) was used. The title compound was obtained as a dark red solid in 94 %yield (83 mg, 0.08 mmol). mp >260°C. ^1H NMR (300 MHz, DMSO-d_6) δ 8.91 (d, J = 6.0 Hz, 3H), 8.51 (m, 6H), 8.00 -7.88 (m, 12H), 7.54 (d, J = 16.0 Hz, 3H), 7.21 (d, J = 8.5 Hz, 6H), 4.37 (s, 9H). ^{13}C NMR (75 MHz, DMSO-d_6) δ 152.6, 148.6, 145.9, 145.6, 144.0, 142.4, 130.3, 130.1, 129.5, 126.0, 125.4, 124.6, 123.0, 115.6, 46.0. LR-MS (ESI-MS): m/z 200.2 $[M-3I^-]^{3+}$ (100%), 292.3 $[M-Me-3I^-]^{2+}$ (35%), 363.3 $[M-2I^-]^{2+}$ (30%). HRMS (ESI+): m/z calculated for $[C_{42}H_{39}N_4]^{3+}$: 199.7725; found: 199.7704.

^1H and ^{13}C NMR spectra of TP2-^{N}Pyo, TP3-^{N}Pyo, TP2Pyo and TP3Pyo are shown in Fig. S8.

Colocalization experiments. TPAs (2-µM for TP2Bzim and TP2Pyo; 6-µM for TP3Py and TP3Pyo) were pre-incubated for 2h with living HeLa cells before observation by confocal microscopy. All TPAs were excited at 458 nm (emission slit settings: 530-690 nm for TP3Bzim and TP2Bzim; 560-720 nm for TP2Py, TP3Py, TP2Pyo and TP3Pyo). For colocalization experiments with mitochondria, TPA-treated HeLa cells were washed with PBS buffer (phosphate-buffered saline, pH 7.5, Gibco®) and further incubated with either 20-nM MitoTracker® Green FM or 50-nM MitoTracker® Red FM (Invitrogen) for 30 min. For TP3Py, TP2Pyo, TP3Pyo (red emission) and for TP2Bzim (green emission), MitoTracker® Green FM (excitation: 488 nm/emission slit: 500-550 nm) and MitoTracker® Red FM (excitation: 633 nm/emission slit: 650-700 nm) were used, respectively. For labelling late endosomes, the Cerulean protein (excitation: 458 nm/emission slit: 465-510 nm) was expressed in Hela cells as a fusion to Rab7, a small GTPase associated with late endosomes, by using a lentiviral shuttle vector expressing the *Cerulean-rab7* fusion gene as previously described[2].

Colocalization experiments in the presence of endocytosis inhibitors: colocalization between TP2Py or TP3Bzim and mitochondria was performed in living HeLa cells in the presence of various endocytosis inhibitors (30-µM chlorpromazine, 80-µM dynasore, 5-µM cytochalasin D or 100 µM-genistein). HeLa cells were pre-treated with 20 nM MitoTracker® Green FM (used for TP2Py) or 50 nM MitoTracker® Red FM (used for TP3Bzim) for 30 min. The cells were then washed with PBS buffer and incubated either with the endocytosis inhibitor (inhibitor-treated samples) or with the solvent used to dissolve the inhibitor (control) for 30 min, followed by incubation of TPA (2 µM) for 90 min. The cells were then washed with PBS buffer (x3) for 5 min, to remove any extracellular inhibitor or TPA, before imaging with a SP8 confocal microscope (Leica MicroSystem) (z-step 0.5 µm). Excitation and emission slit settings are indicated in the legend of Fig. S7. The quantitative evaluation of colocalization was performed by using the Manders' Colocalization Coefficients (MCC): tM1 and tM2 which correspond to the fraction of red species in compartments containing green species and the fraction of green species in compartments containing red species, respectively[3,4]. tM1 and tM2 were calculated using the Coloc 2 plugin in Fiji/ImageJ[5] and are presented as mean±SD values from 35 measurements for each condition (7 slices x 5 cells).

Quantitative imaging analysis of TPA fluorescence intensity in cells treated or not with endocytosis inhibitors. Images were treated with the Fiji version of the image processing software ImageJ[5]. The normalized fluorescence intensity per volume unit in a given cell was calculated from the intensity measured in a predefined surface area (region-of-interest) and integrated along the z-stack direction (z-step of 0.5 µm). For each condition, three independent experiments were performed. ≈50 cells were analysed per experiment, giving the median fluorescence intensity (MFI). The uptake percentage in the presence of a given inhibitor was calculated as follow: %(uptake) = (MFI_inhibitor-treated cells / MFI_untreated cells) x100. Finally, results correspond to mean±SD values from the three independent experiments.

Supplementary References

1. Dumat, B. *et al.* DNA switches on the two-photon efficiency of an ultrabright triphenylamine fluorescent probe specific of AT regions. *J. Am. Chem. Soc.* **135**, 12697-12706 (2013).
2. Chennoufi, R. *et al.* Mitochondria-targeted triphenylamine derivatives activatable by two-photon excitation for triggering and imaging cell apoptosis. *Sci. Rep.* **6**:21458 (2016).
3. Manders, E. M. M., Verbeek, F. J. & Aten, J. A. Measurement of colocalization of objects in dual-colour confocal images. *J. Microscopy* **169**, 375–382 (1993).
4. Dunn, K. W., Kamocka, M. M. & McDonald J. H. A practical guide to evaluating colocalization in biological microscopy. *Am. J. Physiol. Cell Physiol.* **300**, 723–742 (2011).
5. Schindelin, J. *et al.* Fiji: an open-source platform for biological-image analysis. *Nat. Methods* **9**, 676-682 (2012).
